# Ribosomal DNA copy number amplification and loss in human cancers is linked to tumor genetic context, nucleolus activity, and proliferation

**DOI:** 10.1371/journal.pgen.1006994

**Published:** 2017-09-07

**Authors:** Meng Wang, Bernardo Lemos

**Affiliations:** Department of Environmental Health & Molecular and Integrative Physiological Sciences program, Harvard T. H. Chan School of Public Health, Boston, Massachusetts, United States of America; Cleveland Clinic Genomic Medicine Institute, UNITED STATES

## Abstract

Ribosomal RNAs (rRNAs) are transcribed from two multicopy DNA arrays: the 5S ribosomal DNA (rDNA) array residing in a single human autosome and the 45S rDNA array residing in five human autosomes. The arrays are among the most variable segments of the genome, exhibit concerted copy number variation (cCNV), encode essential components of the ribosome, and modulate global gene expression. Here we combined whole genome data from >700 tumors and paired normal tissues to provide a portrait of rDNA variation in human tissues and cancers of diverse mutational signatures, including stomach and lung adenocarcinomas, ovarian cancers, and others of the TCGA panel. We show that cancers undergo coupled 5S rDNA array expansion and 45S rDNA loss that is accompanied by increased estimates of proliferation rate and nucleolar activity. These somatic changes in rDNA CN occur in a background of over 10-fold naturally occurring rDNA CN variation across individuals and cCNV of 5S-45S arrays in some but not all tissues. Analysis of genetic context revealed associations between cancer rDNA CN amplification or loss and the presence of specific somatic alterations, including somatic SNPs and copy number gain/losses in protein coding genes across the cancer genome. For instance, somatic inactivation of the tumor suppressor gene *TP53* emerged with a strong association with coupled 5S expansion / 45S loss in several cancers. Our results uncover frequent and contrasting changes in the 5S and 45S rDNA along rapidly proliferating cell lineages with high nucleolar activity. We suggest that 5S rDNA amplification facilitates increased proliferation, nucleolar activity, and ribosomal synthesis in cancer, whereas 45S rDNA loss emerges as a byproduct of transcription-replication conflict in rapidly replicating tumor cells. The observations raise the prospects of using the rDNA arrays as re-emerging targets for the design of novel strategies in cancer therapy.

## Author summary

The 45S and 5S ribosomal DNA (rDNA) arrays contain hundreds of rDNA copies, with substantial variability across individuals in human populations. Although physically unlinked, the arrays also exhibit joint variation across individual genotypes. However, whether this co-variation in copy number (CN) is universally observed across all tissues is unknown. It also remains unknown if rDNA CN might vary across tissues and in cancer lineages. Here we showed that most cancers undergo coupled 5S rDNA array amplification and 45S rDNA loss, and concerted 5S-45S CN variation in some but not all tissues. The coupled 5S amplification and 45S loss is associated with the presence of certain somatic genetic alterations, as well as increased estimates of cancerous cell proliferation rate and nucleolar activity. Our research uncovers frequent and contrasting changes in rDNA CN in cancers of diverse tissue origin and associated with diverse mutational contexts of tumor suppressors and oncogenes. The observations raise the prospects of using the rDNA arrays as re-emerging targets in novel strategies for cancer therapy.

## Introduction

The ribosomal DNA (rDNA) arrays give origin to the nucleolus, the nuclear organelle that is the site of ribosomal RNA (rRNA) transcription and ribosome biogenesis [1]. The rRNAs constitute the vast majority of cellular RNAs and are encoded from two kinds of tandemly repeated ribosomal DNA (rDNA) arrays [2–5]. The 45S rDNA array is localized on five human chromosomes, is transcribed by RNA polymerase I (Pol I), encodes the 45S rRNAs that are processed into three rRNAs (18S, 5.8S and 28S rRNAs), and organizes the formation of the nucleolus [6]. The 5S rDNA array is exclusive to human chromosome 1, encodes the Pol III transcribed 5S rRNA, and localizes, together with dispersed tRNAs, at the periphery of the nucleolus. Stunningly high transcription of the rRNAs is required to supply ribosomes, essential cellular machines with tightly controlled and strikingly complex biogenesis involving products transcribed by all three RNA polymerases. Indeed, the human ribosome is composed of about 80 cytoplasmic ribosomal proteins (cRP) and four ribosomal RNAs (rRNA; 18S, 5.8S, 28S, and 5S components), responsible for protein production and the translation of all protein-coding mRNAs. Modulation of rRNA synthesis during the cell cycle is critical for cell growth and proliferation [7–9]. To initiate rRNA transcription, proteins such as UBF and TIF are required to facilitate Pol I binding onto the rDNA promoter—a region that includes the upstream control element (UCE) and the core sequence. The protein components of the cytoplasmic ribosomes, cRPs, are co-expressed to ensure stoichiometric balance [10–15]. Moreover, hundreds of other proteins and several snoRNAs are needed for the cleavage, modification, transport, and assembly of rRNAs, and the maturation of ribosomes in the nucleolus and nucleoplasm.

It is thus not surprising that altered ribosomal biogenesis and rRNA regulation has been linked to a variety of human diseases. The dysregulation of cRP genes (cRPGs) can destabilize rRNAs and/or disturb their synthesis [16, 17], or alternatively participate in tumorigenesis through *TP53*-related pathways [18, 19]. Notably, many well established oncogenes and tumor-suppressor genes are directly or indirectly involved in regulating ribosomal biogenesis and/or nucleolus function [20–22]. For example, proteins in the retinoblastoma (*RB*) family as well as TP53 and MDM2/4 regulate nucleolar function and rRNA synthesis: they can restrict rRNA synthesis by interacting with UBF and repressing Pol I activity [23–25]. Also, the proto-oncogene product, c-Myc, can target cRPGs as well as other nucleolar proteins such as nucleophosmin (NPM) and Nucleolin [26–28]. Collectively, the observations imply an integrated network of cellular functions that is coherently modulated and is centered on rDNA/nucleolus maintenance, rRNA expression, and ribosome biogenesis.

Both the 5S and 45S rDNA arrays display remarkably variable copy number (CN), ranging from tens to hundreds of copies among eukaryotes [3, 5, 29–32] and displaying a 10-fold variation among individuals in human populations [3, 5, 30]. Surprisingly, copy number of the 5S and 45S rDNA arrays in human blood is positively correlated across genotypes in spite of 5S and 45S array location in separate chromosomes and lack of sequence homology [3]. The observations indicated that copy number in both arrays is jointly modulated and suggested a general mechanism of concerted copy number variation (cCNV) that is likely to operate in other tissue types to maintain balanced 5S and 45S rRNA. Notwithstanding, only a fraction of the rDNA units are transcribed per cell [33, 34] and alternative mechanisms exist to modulate 45S rRNA supply. In addition, rDNA CN itself might contribute to nucleolar function, providing a critical mechanism to maintain genome stability [35] and exerting genome-wide consequences to gene regulation [30, 36]. Finally, a number of seminal ultra-structural studies documented alterations in nucleolar morphology during carcinogenesis [37–40]. In spite of the manifold cellular roles of the rDNA array, the manifestation of concerted copy number variation of the 5S and 45S arrays has not been examined in human tissues other than blood. Furthermore, the role of rDNA CN and altered ribosomal function in human cancers remain to be characterized. Here we ascertained rDNA copy number in several tissues from hundreds of individuals and cancer genomes. We applied new methods and corrections for aneuploidy and sequencing batch to provide a comprehensive portrait of rDNA variation in cancer and normal tissues. The data were integrated with genetic context to reveal associations between specific somatic alterations and somatic rDNA CN changes, as well as the impact of nucleolar activity and proliferation rate.

## Results

### Estimating rDNA copy number in human cancers

**Fig 1:**
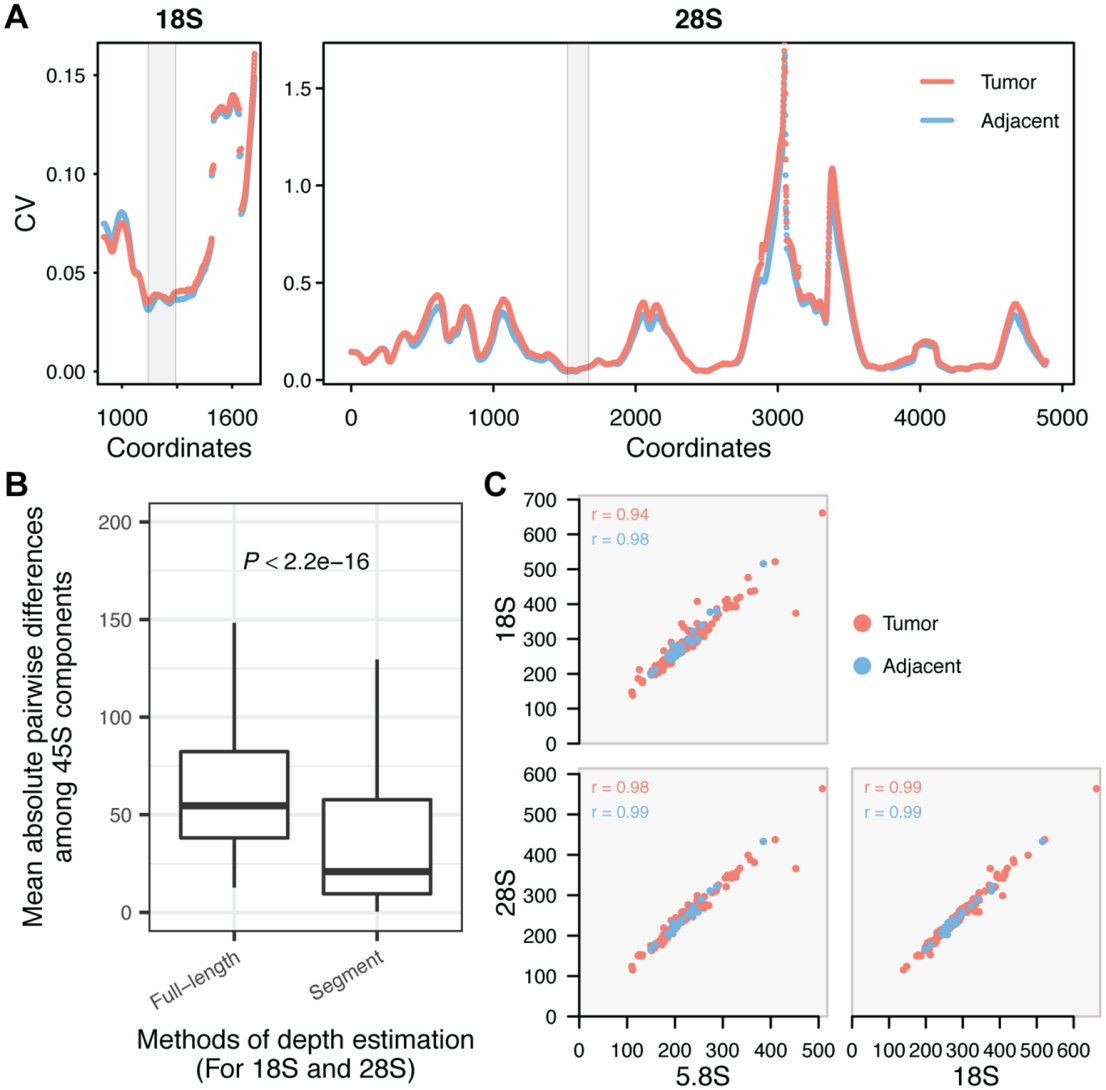
Estimating rDNA copy number. (A) For each 150bps window along the rDNA array reference, we calculated the coefficient of variation (CV) of the average depth among samples. The x-axes indicate the starting coordinate for each window on 18S (901–1871) and 28S (whole length). LUAD tumor and adjacent tissues have largely consistent CV across 18S and 28S sequences. The grey regions highlight the windows selected to assess CN in the 18S and 28S components. (B) The differences in copy number among the three 45S components in each sample were calculated as the mean of their absolute pairwise difference. Such differences are significantly smaller when using the selected segments than using the full length of the rDNA shown in (A) for 18S and 28S. (C) Nearly perfect pairwise Pearson correlations between 45S components. BLCA is displayed as an example. Copy number estimates are corrected for batch and ploidy.

Ribosomal RNAs are transcribed from the 45S and 5S rDNA repeats, both of which display ~10-fold variation in copy number within populations. Here we used whole-genome sequencing data (WGS) from the TCGA to ascertain rDNA CN variation across 946 samples (721 tumor and 225 adjacent normal tissue) representing six cancer types with the largest numbers of individuals with paired tumor and adjacent normal tissue [i.e., bladder urothelial carcinoma (BLCA), lung adenocarcinoma (LUAD), lung squamous cell carcinoma (LUSC), kidney renal clear cell carcinoma (KIRC), head and neck squamous cell carcinoma (HNSC), and stomach adenocarcinoma (STAD)] (Table 1). We adopted a computational approach developed in earlier studies [3, 30] with important modifications to estimate rDNA CN in tumors. Briefly, rDNA CN was estimated by dividing the average depth of each component (18S, 5.8S, 28S and 5S) by the average depth of the selected background, i.e. single copy exons and introns residing on chromosomes bearing rDNA arrays. To diminish the effect of heterogeneity within 18S and 28S sequences, we identified segments with the smallest coefficient of variation in read depth across sites to represent the two components (Fig 1A). Compared to using the full length of each component, the segment method indeed yielded smaller differences in CN estimates among 18S, 5.8S and 28S (Fig 1B), an outcome favoring the latter method because the three 45S components are a functional repeat unit. Furthermore, tumor aneuploidy is rampant [41, 42] and can be a major confounder in rDNA CN estimates. Hence, we accounted for ploidy variation in tumors using estimates of per gene copy number amplification/loss from the FireBrowse portal, which were ascertained by the GISTIC 2.0 pipeline [43] using the genome-wide SNP array data. We confirmed extensive ploidy changes in tumors (S1 Fig), which suggested the need for correction in rDNA CN estimates. Tumor aneuploidy and gene amplification/loss will reflect on the segment sequencing depth, such that a positive correlation between ploidy estimates and read depth is expected. Indeed, we did observe such relationship in tumor samples (S2A Fig), which supported our methodology and underscored the necessity of correction for aneuploidy. Intriguingly, we also detected significant batch effects of TCGA plate ID on estimates of rDNA CN (S2B Fig and S1 Table), which are reminiscent of batch effects observed in recent mtDNA copy number estimates for tumors [44]. Here we specifically controlled for sequencing batch using linear regression model separately for each cancer type (see Methods). Finally, we identified rDNA reads by “slicing” of (BAM) files generated by the TCGA consortium (see Methods). The approach had a marginal effect on rDNA estimates, when compared to *de novo* identification of rDNA reads by processing and mapping of raw reads to rDNA references (S2C Fig).

**Table 1:** Cancer types with rDNA CN estimated in this study.

Cancer type	TCGA ID
**Bladder Urothelial Carcinoma**	BLCA
**Lung adenocarcinoma**	LUAD
**Lung squamous cell carcinoma**	LUSC
**Stomach adenocarcinoma**	STAD
**Kidney renal clear cell carcinoma**	KIRC
**Head and Neck squamous cell carcinoma**	HNSC
**Ovarian Serous Cystadenocarcinoma**	OV
**Breast Invasive Carcinoma**	BRCA
**Liver Hepatocellular Carcinoma**	LIHC

Due to their close physical linkage, dosage (copy number) of the 45S rDNA components (18S, 5.8S and 28S rDNA) is expected to be congruent with each other, although not necessarily identical due to non-canonical and truncated rDNA units [45, 46]. Indeed, we observed positive correlations among 18S, 5.8S and 28S components in adjacent normal tissue of patients with all cancer types (Fig 1C and S3 Fig; *P* < 0.005 for all samples). Upon removal of aneuploidy, sequencing batch confounders, and use of selected segments, we observed that the 45S components had nearly perfect correlations among each other in LUAD, STAD, BLCA, and HNSC (Fig 1C and S3 Fig; *P* < 0.0001 for all samples). The analyses improved on earlier methods for computing rDNA CN estimates [30] and showed that rDNA CN can be reliably ascertained in both cancer and normal tissues.

### Concerted copy number variation is not universally manifested in all tissues

**Fig 2:**
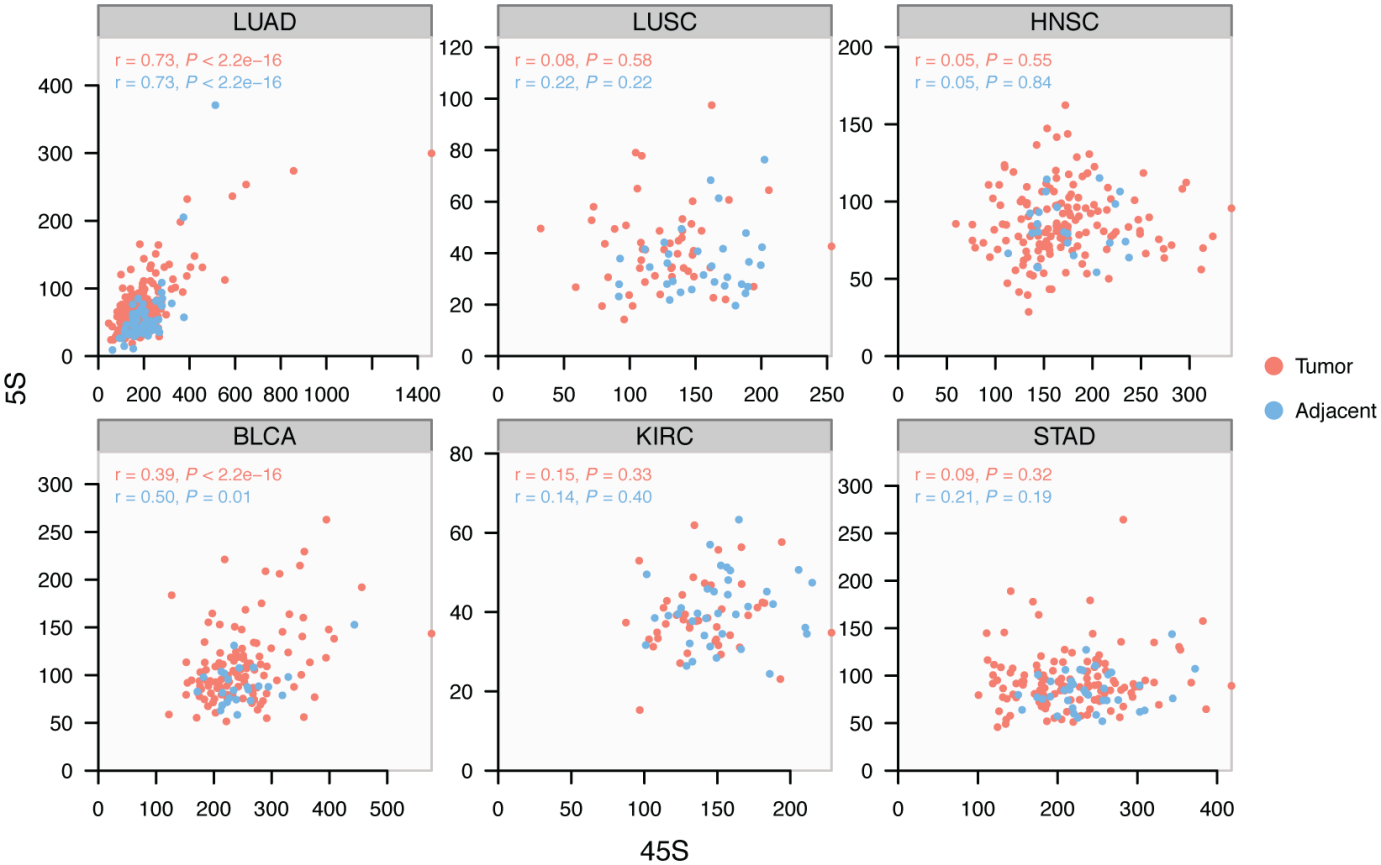
Variable manifestation of concerted copy number variation (cCNV) across tissues. Pearson correlation between the 5S and 45S rDNA arrays is variable among tissues. The correlation is significant and similarly manifested in normal and cancer lineages of LUAD and BLCA.

The 5S array is located on an unrelated chromosome and has evolved copy number variation that is tightly correlated with copy number of the 45S rDNA in B-cell derived lymphoblastoid cell lines (LCLs) and whole blood [3]. However, tissue specific copy number control has been reported across diverse eukaryotes [47]. Here we applied the improved methodology and corrections to ascertain rDNA copy number of the 5S rDNA and address whether the 5S and 45S rDNA arrays display cCNV in human solid tissues. We observed that the manifestation of cCNV is variable among rDNA components and across tissues (Fig 2, S3 and S4 Figs). Specifically, we observed strong cCNV for both tumor and adjacent tissues in BLCA and LUAD, although only the 5.8S is significantly correlated with 5S in KIRC and LUSC. On the other hand, cCNV appeared absent in HNSC and STAD. These results suggest that the strength of cCNV is variable across tissues, and that the phenomenon is not universally manifested in all tissues.

### Ribosomal DNA amplification and loss in paired cancer and adjacent normal tissue

**Fig 3:**
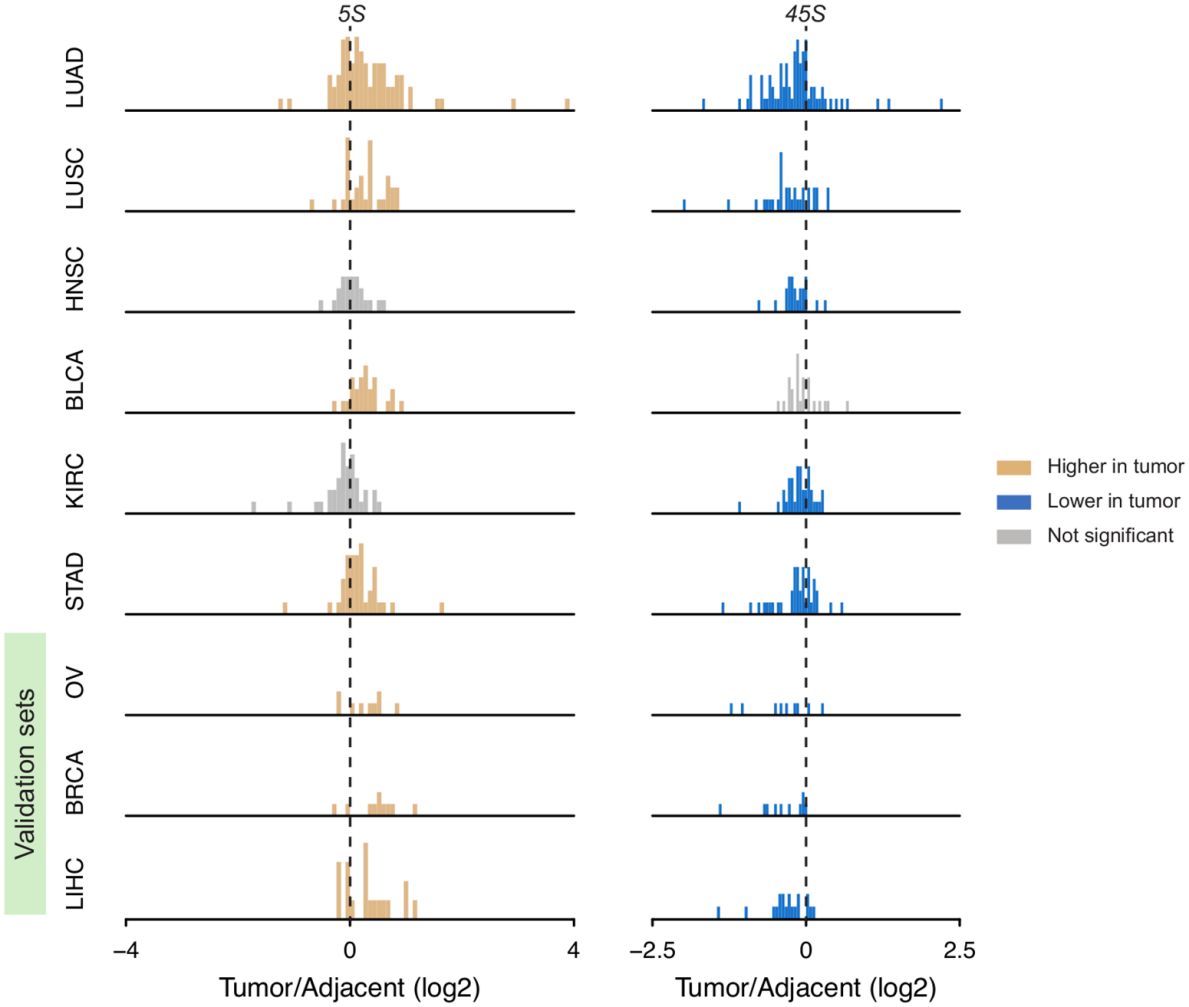
rDNA CN amplification and loss between cancer tissue and paired adjacent normal tissue. Yellow and blue denote significant gain or loss in tumors compared with paired adjacent controls (one-sided paired Wilcoxon rank sum test *P* < 0.05 for OV and < 0.01 for others). There are 74, 32, 22, 24, 35, 38, 9, 10 and 19 informative patients for LUAD, LUSC, HNSC, BLCA, KIRC, STAD, OV, BRCA and LIHC respectively. OV, BRCA and LIHC were used as validation sets.

We compared paired tumor and adjacent tissue within an individual to obtain estimates of rDNA fold-change amplification and loss in cancer lineages. To minimize confounders due to plate ID, analyses with tumor-adjacent comparisons only included 225 patients with both tumor and adjacent tissue sequenced in the same plate. Intriguingly, we found a mild but significant depletion of 45S rDNA in 5 out of 6 cancer types relative to adjacent normal tissue (Fig 3 and S2 Table). The exception is BLCA showing only a slight but not significant reduction in 45S rDNA. The events of 45S loss are especially salient in LUAD with 73% (54/74) of all paired-adjacent contrasts revealing loss events, with 13% (7/54) of all loss events showing less than 60% of the copy number value in the adjacent tissue. On the other hand, we observed amplification of the 5S rDNA array in 4 cancer types, regardless of loss in the 45S components (Fig 3). Interestingly, LUAD stood out with 68.9% (51/74) of the cases showing amplification events, with 47.1% (24/51) of them reflecting a >40% increase in 5S rDNA copy number. Notably, cCNV between 5S and 45S in LUAD tumors remains detectable, in spite of their contrasting patterns of alterations. To verify the patterns of 5S and 45S changes in a validation set, we further analyzed 3 cancer types [ovarian serous cystadenocarcinoma (OV), breast invasive carcinoma (BRCA) and liver hepatocellular carcinoma (LIHC)] (Table 1). Again, we observed significant amplification of 5S and loss of 45S in all 3 types, although fewer tumor-adjacent pairs are available for them (Fig 3 and S2 Table). We conclude that 5S rDNA amplification and 45S loss are recurrent events in cancer lineages of various origins.

### Cancer somatic copy number alterations (SCNAs) couple 5S expansion and 45S loss

**Fig 4:**
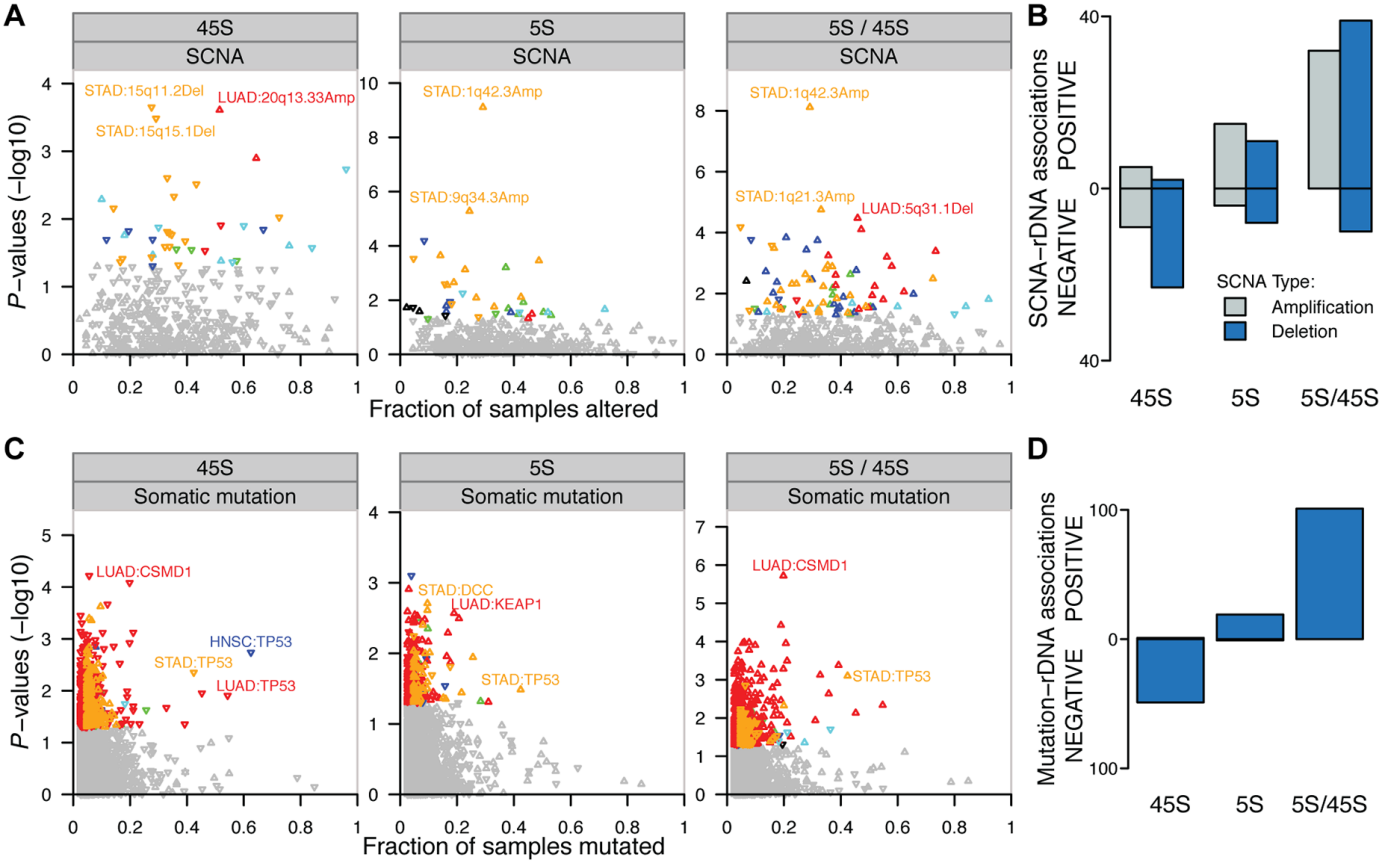
Association between genetic context and rDNA CN alterations. (A) Associations between copy number alterations (SCNA) and 5S CN, 45S CN and 5S/45S ratio. (B) Significantly associated rDNA-SCNA pairs (*P* < 0.05) are preferentially implicated in greater 45S loss and greater 5S rDNA amplification (*P* < 0.05, binomial test). (C) Association between somatic mutations and 5S CN, 45S CN and 5S/45S ratio. (D) Significantly associated mutation-rDNA pairs (*P* < 0.05) are almost exclusively implicated in greater 45S loss and greater 5S rDNA amplification (*P* < 0.001, binomial test) in LUAD. For (B, C) Y-axis show the P-values for the associations between the SCNA or gene mutation event and 45S CN, 5S CN and 5S/45S ratio. rDNA associations were colored according to cancer type (*P* < 0.05). The up/down direction of triangles indicates that the somatic alteration is associated with increased or decreased CN or 5S/45S ratio. The X-axis shows the fraction of patients with the non-silent gene mutation or focal SCNAs (ploidy cutoff > 2.1 for amplification and < 1.9 for deletion).

Multiple mutations are involved in cancer development. These include mutations in genes that control genome stability and that can trigger other downstream mutation events [42, 48, 49]. Here we investigated the impact of genomic variations on rDNA CN in tumors. To address the issue, we cross-referenced our estimates of rDNA CN with estimates of local somatic copy number alterations (SCNAs) as ascertained by the TCGA consortium. The data allowed us to stratify tumors by the presence or absence of a SCNA, and examine the association between a SCNA and CN of the 5S or 45S rDNA. As a result, we observed 39 and 38 SCNA events significantly correlated with 5S and 45S CN, respectively, in at least one cancer type (Fig 4A and S3 Table, *P* < 0.05). Reassuringly, the strongest association for 5S is its positive correlation with 1q42.3 amplification in STAD (Fig 4A). This association is expected because the 5S rDNA resides within the 1q42 segment. Besides 1q42 amplification, most other significant rDNA-associated SCNAs are physically unlinked to either the 45S or the 5S rDNA array. For example, 9q34.3 amplification is significantly associated with the accumulation of 5S (Fig 4A, linear regression’s coefficient = 31.15 and *P* = 5.23e-6), whereas 15q11.2 deletion is associated with 45S loss in STAD (Fig 4A, coefficient = -91.86 and *P* = 0.00022). The *MDM2* gene locus, whose protein product links 5S rRNA to TP53 homeostasis [50], is also significantly amplified in tumors (S5 Fig), although no clear correlation was observed between the *MDM2* locus and rDNA CN in our analysis. Most notably, the majority of the significant SCNA-45S associations are implicated in greater 45S loss along the cancer lineages (Fig 4B). Specifically, 9/14 amplification events and 23/25 deletion events resulted in greater 45S rDNA loss (binomial test for total, *P* = 7.025e-05). On the other hand, the majority of significant SCNA-5S associations are implicated with events of 5S amplification along the cancer lineage (Fig 4B). Specifically, 15/19 amplification events and 11/19 deletion events resulted in greater 5S amplification (*P* = 0.034 for total). Because of the frequent co-occurrence of 5S gain and 45S loss, we correlated SCNAs with 5S / 45S ratios. In agreement with our expectations, we found that nearly all of the statistically significant rDNA-SCNA associations are implicated in higher 5S/45S ratios in the tumor lineage (Fig 4B, 71/81, *P* = 1.799e-12), regardless of whether the focal SCNA was a deletion or a duplication event.

### Dosage of the 5S rDNA is increased through both multicopy 5S array expansion and 1q42 segmental duplication

**Fig 5:**
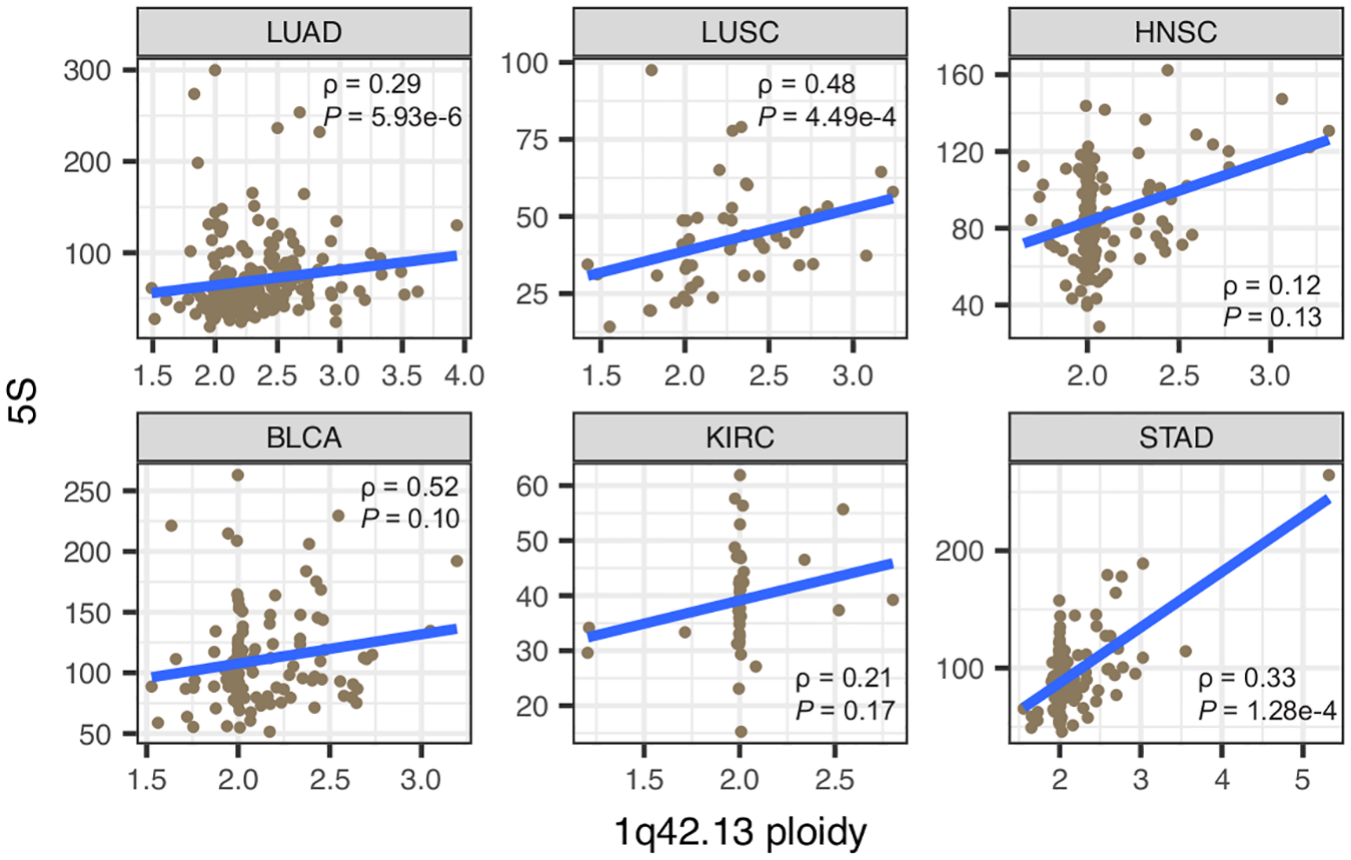
Increased 1q42.13 ploidy partially explains increased 5S rDNA CN in cancers. In each cancer type, all available tumors were included. Spearman’s rank correlation was used.

**Fig 6:**
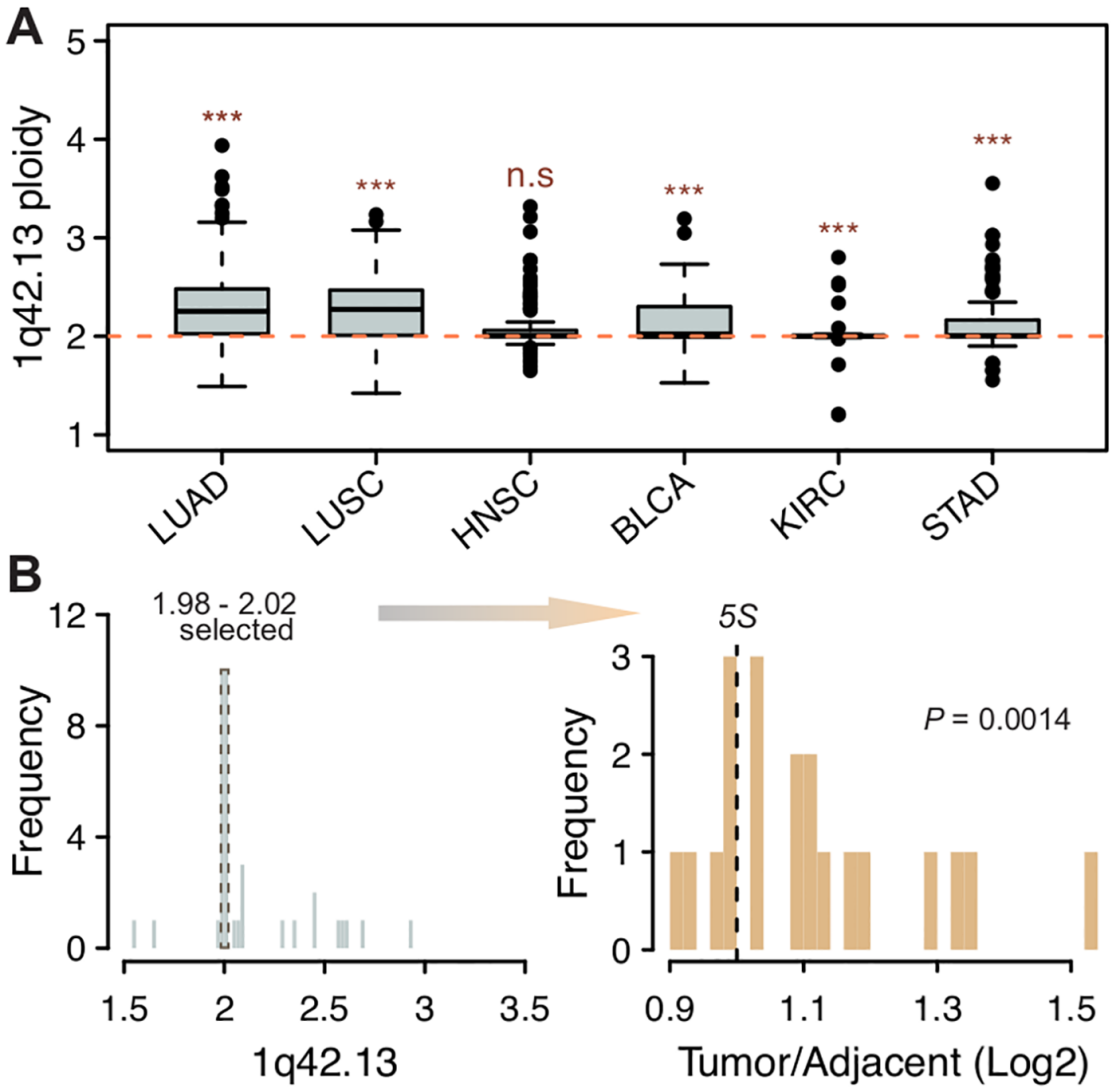
The 5S rDNA is increased through 1q42 segmental duplications and 5S array expansion. (A) Most cancers displayed significantly increased 1q42.13 ploidy. (B) Significant 5S CN amplification was still observed in STAD when only considering patients that are closest to diploidy at 1q42.13 (P-value from one-tailed Wilcoxon rank sum test). Only patients with 5S rDNA CN estimated for both tumor and adjacent tissues are shown in B.

To further understand the mechanisms for 5S amplification, we used two genes localized immediately upstream and downstream of the 5S array (*RNF187* and *RHOU*), as a proxy to address segmental duplications involving the 1q42.13 region. First, we observed significant positive correlations between estimates of 1q42.13 ploidy and 5S rDNA CN in LUAD, LUSC and STAD (Fig 5). Second, we observed that the 1q42.13 segment is significantly amplified in all but one cancer type (Fig 6A). This suggests that amplification of the 1q42 segment is a common and recurrent mechanism causing increased 5S CN across distinct cancers. It is noteworthy that the correlation is not significant in the other 3 cancer types, which raises the possibility that other oncogenes within/near this region, such as the *PCaP* (Predisposing for Cancer Prostate) locus on 1q42.2–43 [51] and *SMYD3* on 1q44 [52], could be contributing to the selective advantage of tumor lineages. On the other hand, recombination within the 5S rDNA array could provide an alternative mechanism contributing to 5S rDNA amplification. To address the issue we examined patients for which ploidy estimates at 1q42.13 is closest to diploidy (1.98–2.02). In agreement with our hypothesis that 5S array recombination is an important mechanism for expansion, significant 5S amplification was still observed in individuals for which 1q42 ploidy did not increase (Fig 6B, one-tailed Wilcoxon rank sum test, *P* = 0.0014, STAD). In conclusion, the data suggest that both 1q.42 segmental duplications as well as local 5S rDNA array expansion contribute towards increased 5S rDNA dosage in cancer lineages.

### *TP53* mutations couple 5S expansion and 45S loss

We next examined somatic mutations associated with rDNA CN amplification or loss in each cancer type. Specifically, we identified tumors containing non-silent somatic mutation(s) in protein-coding genes and compared their rDNA CN amplification or loss events with those tumors from individuals without cancer mutations in the focal gene. We examined a total of 17,035 genes containing somatic mutations with 1,481 of which being present in ten to a few hundred individuals in at least one cancer type. Strikingly, we found that *TP53* mutations are negatively associated with 45S CN in STAD, HNSC (*P* < 0.005, Wilcoxon rank sum test) and LUAD (*P* = 0.012, Wilcoxon rank sum test) (Fig 4C and S4 Table). On the other hand, most LUSC tumors show *TP53* mutations while most KIRC tumors do not show *TP53* mutations, leading to low statistical power for *TP53*-rDNA association in both cases. Presence of *TP53* mutation is also associated with 5S amplification in STAD (Fig 4C, *P* = 0.033). We also identified somatic mutations on other tumor suppressor genes significantly associated with rDNA CN in tumors. For example, *CSMD1* is associated with decreased 45S in LUAD, while *KEAP1* and *DCC* are associated with increased 5S in LUAD and STAD, respectively. Overall, the associations between mutation presence and rDNA CN are particularly apparent in LUAD, the cancer with the largest sample size. In this case, the associations between mutation presence and 45S rDNA CN are predominantly negative (Fig 4D, 49/50, binomial test *P* = 9.059e-14), whereas the association between mutation presence and 5S CN are predominantly positive (19/20, *P* = 4.005e-5). The positive associations for the 5S rDNA reinforce the notion that tumors might be selected for 5S rDNA CN increases, possibly to supply the cell with increased protein synthesis and proliferative capacity. Finally, the contrasting direction of the gene-5S and gene-45S associations is most evident when examining the ratio of 5S / 45S rDNA in the presence of these somatic mutations: all 101 significant gene-rDNA (5S/45S ratio) associations are positive (*P* < 2.2e-16). This indicates a higher 5S/45S ratio in the presence of specific somatic mutations.

To increase statistical power, we combined all tumors in a pan-cancer analysis of gene-rDNA association. For each gene, we applied an ANOVA to compare 45S, 5S or their ratio between the groups with and without the mutation, with cancer types as a covariate. We observed dozens of genes significantly associated with 45S, 5S, and their ratio, respectively (S5 Table). In this analysis *TP53* mutations emerged again as one of the top candidates associated with significantly lower 45S (*P* = 0.0015), as well as significantly higher 5S / 45S ratio (*P* = 5.24e-9). Among genes associated with the 5S we find that 89.5% (128/143) of them displayed positive associations, indicating higher 5S rDNA CN in the presence of the mutation. In this pan-cancer analysis, >95% of all significant associations between the presence of the somatic mutation and the 5S / 45S ratio are positive. Additionally, the *TP53* gene has a rich spectrum of somatic mutations, which may contribute to cancers in different ways [53]. We then asked if rDNA CN variation is associated with specific *TP53* mutations. To address this, we did a pan-cancer analysis for *TP53* mutations that recurred in at least 5 patients. As a result, we identified 6 and 3 mutations associated with 5S / 45S ratio under cutoff *P* < 0.1 and cutoff *P* < 0.05, respectively (Table 2). All these mutations are missense and all of them are associated with increased 5S / 45S ratio, as expected from our previous analysis with every *TP53* mutation merged.

**Table 2:** Pan-cancer linear associations between somatic *TP53* mutations and the 5S / 45S ratio*.

Mutation	Coefficient	*P* value	Mutated patients (696 in total)	Annotation (for transcript ENST00000269305.4)
All mutations merged	0.0705	5.24E-09	360	-
chr17:7577085C>T	0.232	0.00777	5	E285K
chr17:7578461C>A	0.217	0.0127	5	V157F
chr17:7578190T>C	0.191	0.0276	5	Y220C
chr17:7578406C>T	0.114	0.0526	11	R175H
chr17:7578403C>A	0.168	0.0526	5	C176F
chr17:7578271T>C	0.127	0.0873	7	H193R
chr17:7577120C>T	0.0903	0.126	11	R273H
chr17:7578212G>A	-0.131	0.133	5	R213* (nonsense)
chr17:7578263G>A	-0.111	0.163	6	R196* (nonsense)
chr17:7577121G>A	-0.0683	0.248	11	R273C
chr17:7578275G>A	0.0802	0.315	6	Q192* (nonsense)
chr17:7577099C>A	-0.0331	0.705	5	R280I
chr17:7578394T>C	-0.0268	0.761	5	H179R
chr17:7577548C>T	-0.0221	0.781	6	G245S
chr17:7579312C>G	0.0152	0.837	7	Splice_site
chr17:7578454G>A	-0.0144	0.869	5	A159V
chr17:7577538C>T	-0.00413	0.927	19	R248Q
chr17:7577547C>A	-0.00395	0.964	5	G245V

*The association of 5S/ 45S ratio with each specific *TP53* mutation that recurred in ≥ 5 patients is shown. The first row shows the outcome when all non-silent somatic *TP53* mutations are merged. Cancer types were used as a covariate. A positive coefficient indicates a higher 5S /45S ratio in the presence of the mutated *TP53* allele.

### Increased nucleolar activity and proliferation rates link 5S rDNA amplification and 45S rDNA loss

**Fig 7:**
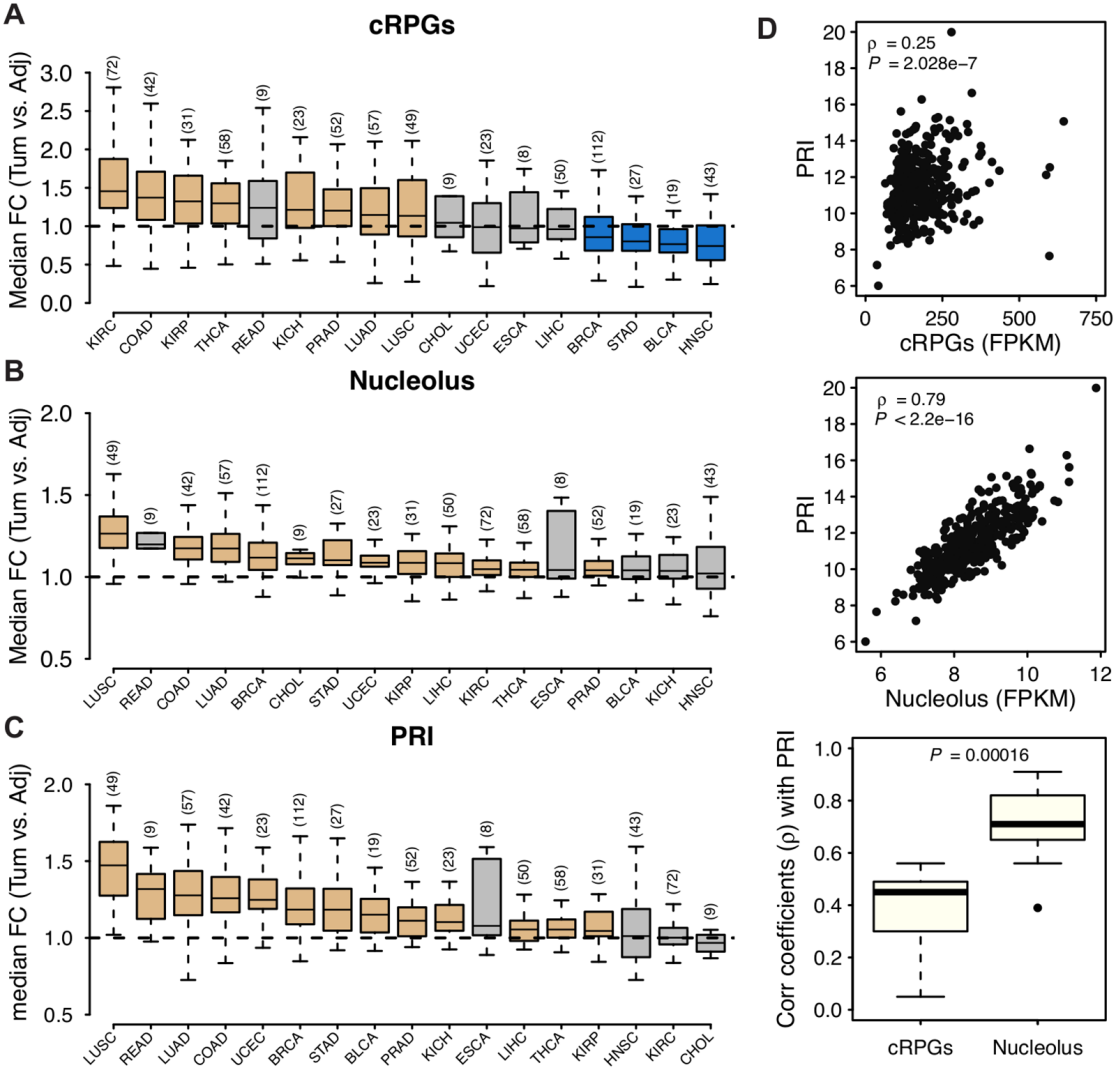
Nucleolar gene expression and proliferation in cancer-adjacent tissue pairs. (A-C) Changes in the level of expression of (A) cytoplasmic ribosomal protein genes (cRPGs), (B) nucleolar genes as well as (C) PRI in tumors relative to their normal adjacent tissue within each individual. Seventeen cancer types with RNA-seq data in ≥ 5 tumor-adjacent pairs were shown, with sample sizes in brackets. Yellow and blue indicate significant up- and down- regulation in tumors compared with paired adjacent control tissue (Wilcoxon rank sum test *P* < 0.01), respectively. Non-significant changes are shown in grey. (D) PRI is more strongly correlated with the expression of nucleolar genes than with the expression of cRPGs. The upper two panels show the correlations of cRPGs and nucleolar genes with PRI in BLCA as an example, while the lower panel summarizes the Spearman’s correlation coefficients in all 17 cancer types (*P* from paired Wilcoxon rank sum test). COAD, colon adenocarcinoma; KIRP, kidney renal papillary cell carcinoma; THCA, thyroid carcinoma; READ, rectum adenocarcinoma; KICH, kidney chromophobe; PRAD, prostate adenocarcinoma; CHOL, cholangiocarcinoma; UCEC, uterine corpus endometrial carcinoma; ESCA, esophageal carcinoma. Other abbreviations are as in Table 1.

We hypothesize that contrasting rDNA CN variation may reflect increased nucleolar activity as well as rapid tumor cell proliferation. To address the issues we first examined the median expression of cRPGs and nucleolar genes across tumors and adjacent tissues. cRPG denote genes encoding the protein components of the large and small ribosomal subunits (GO:0022625 and GO:0022627; S6 Table). Nucleolar activity is defined as the median expression of the 799 genes belonging to the cellular component “nucleolus” (GO:0005730; S6 Table). We compared tumor tissues relative to their adjacent normal, and indeed observed significantly increased ribosomal and nucleolar gene expression for most cancers (Fig 7A and 7B); the observation is concordant with reports of increased ribosome biogenesis in cancers [54]. We further calculated a proliferation index (PRI) in cancer and adjacent tissues. PRI is based on the expression of 793 genes (denoted as ‘YW’ set) which were recently identified by positive correlation with proliferation rate across 60 cancer cell lines [55]. A larger PRI indicates higher proliferation in the cell lineage. We also measured PRI using a second set of 350 genes implicated in cell proliferation rates [56] (denoted as ‘RS’ set). While the YW and RS sets are mostly independent of each other (only 74 genes are shared), the PRI estimates using the two sets are highly positively correlated (S6A Fig, Spearman’s **ρ** = 0.53–0.89, *P* < 2.2e-16). Furthermore, we observed significantly increased PRI for almost all cancer types (Fig 7C and S6B Fig) with LUSC, LUAD, and STAD among the top cancers showing the largest PRI increases in cancer relative to adjacent normal. Notably, higher PRI is associated with lower survival probability (S7A and S7B Fig, also in [55]), as well as more advanced tumor stages (S7C and S7D Fig). Finally, we observed that the expression of nucleolar genes is an excellent predictor of PRI whereas the expression of cRPGs is a poorer predictor of PRI (Fig 7D and S6C Fig) across several cancers; importantly, the vast majority of genes used to calculate PRI are neither nucleolar nor cRPGs (S8 Fig).

**Fig 8:**
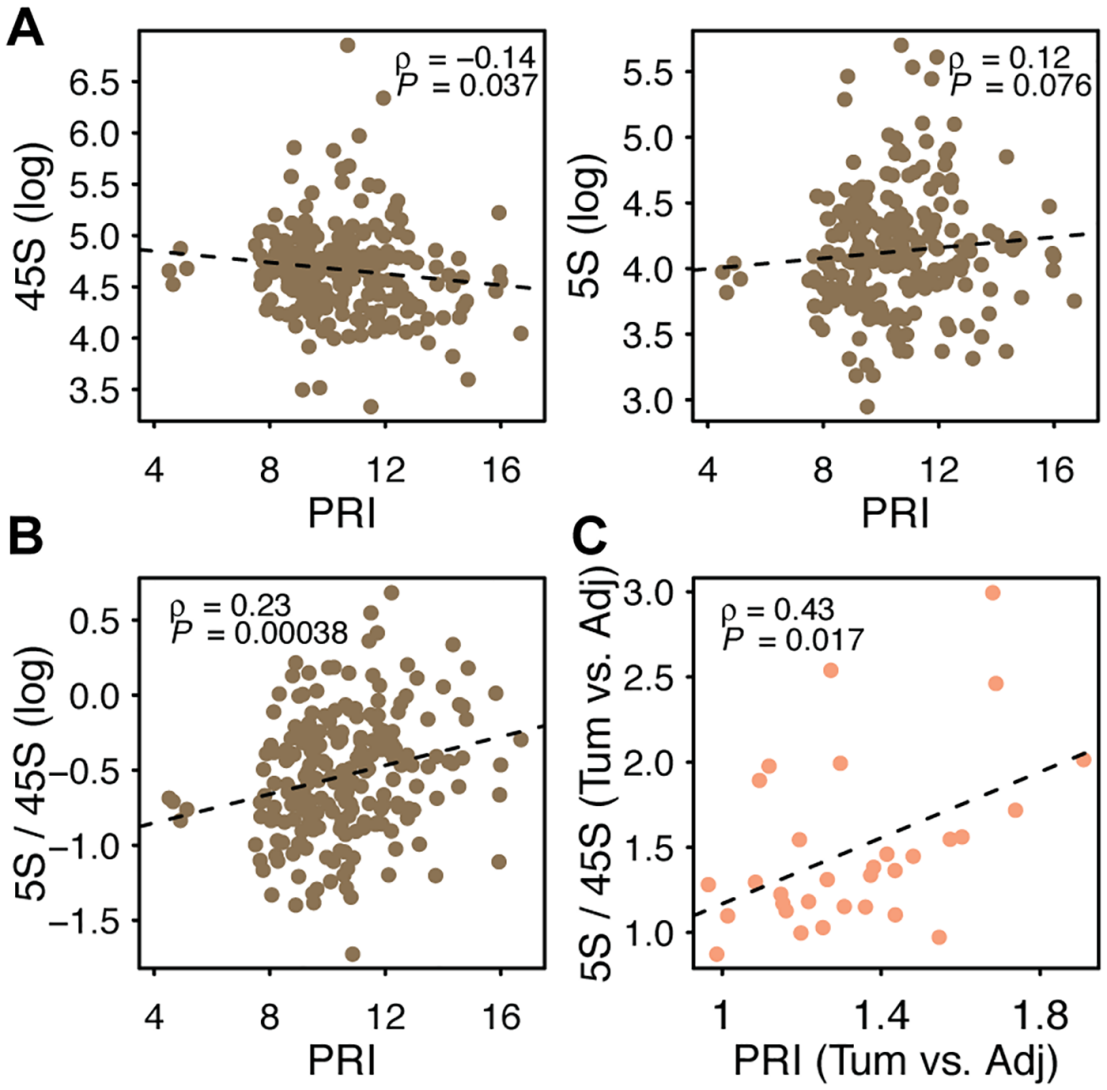
Associations between tumor proliferation and rDNA copy number. (A-B) Spearman rank correlations between PRI (YW gene set) and 45S CN, 5S CN and 5S/45S ratio in LUAD tumor samples. (C) Changes in tumor PRI relative to normal adjacent tissue are positively correlated with changes in the 5S/45S ratio between tumor and normal adjacent tissue. The 31 LUAD patients with paired adjacent-tumor data (DNAseq and RNAseq) were used in C.

Next, we focused on LUAD, the cancer with the highest number of adjacent-tumor pairs, to examine the links between tumor proliferation and changes in rDNA CN. In agreement with our hypothesis, we observed that 45S rDNA CN is negatively correlated with PRI (Fig 8A, **ρ** = -0.14, *P* = 0.037; S9A Fig), whereas 5S rDNA CN is positively correlated with PRI (Fig 8A, **ρ** = 0.12, *P* = 0.076; S9A Fig). The 5S / 45S ratio is also positively correlated with PRI (Fig 8B, Spearman’s **ρ** = 0.23, *P* < 0.001; S9A Fig). To verify the pattern, we then selected individuals with rDNA CN and expression data available in both tumor and adjacent tissues. For these individuals, we calculated the relative fold change (FC) in PRI in tumor relative to its adjacent tissue, and correlated it with the FC in 5S rDNA CN, 45S rDNA CN or their ratio for the same patients. Thirty-one patients are eligible for this analysis. As expected, we observed a negative correlation between increases in proliferation and decreases in 45S CN (S10 Fig, Spearman’s **ρ** = -0.35, *P* = 0.053; S9B Fig). Similarly, the data show a positive correlation between increases in proliferation and increases in the 5S / 45S ratio (Fig 8C, **ρ** = 0.43, *P* = 0.017; S9B Fig). The higher the increase in proliferation observed in a cancer lineage relative to the normal tissue within an individual the greater the loss in 45S rDNA and the higher the change in 5S/45S ratio. We also observed qualitatively similar pattern for LUSC and HNSC using both PRI sets (S7 Table). The expression of nucleolar genes is also negatively associated with 45S rDNA CN (**ρ** = -0.23, *P* = 0.00038) and positively associated with the 5S/45S ratio (**ρ** = 0.27, *P* = 3.08e-5). Finally, the negative correlation of *TP53* mutation with 45S, and positive correlations of *TP53* mutation and PRI with 5S / 45S ratio remain significant even when they were analyzed together in a multivariate model (S8 Table). Collectively, our observations raise the hypothesis that increased proliferation is facilitated by greater 5S rDNA dosage, whereas 45S rDNA loss emerges as a byproduct of transcription-replication conflict in rapidly proliferating cells with enhanced nucleolar activity.

## Discussion

Here we addressed variation in rDNA copy number across hundreds of individuals, tissue types, and within cancer lineages. The data revealed coupled 5S rDNA amplification and 45S rDNA loss in cancer genomes relative to paired adjacent normal tissue, with rDNA changes that are associated with somatic mutations. For instance, coupled 5S gain / 45S loss are particularly salient in lineages with *TP53* inactivation from stomach and lung adenocarcinomas, but can also be observed in head and neck squamous cell carcinoma. The pattern of rDNA gain and loss in cancers happens in the context of large differences in wild-type rDNA copy number across individuals and concerted copy number variation between the 5S and 45S rDNA arrays in some but not all tissue types. Finally, we used global gene expression data to estimate tumor proliferation rates and nucleolar activity and show that they can partially explain coupled 5S rDNA gain and 45S rDNA loss.

It is important to highlight that the coupled events of 5S amplification and 45S loss that we observed in cancer linages is, on average, of small magnitude relative to the breadth of naturally occurring variation in 5S and 45S copy number that is observed across individuals in natural populations. For instance, we have previously shown that ribosomal DNA copy number displayed over 10-fold variation in LCLs from two human populations [3, 30]. Here we also observed a similarly large breadth of variation in rDNA CN in normal solid tissues with 2–10 fold copy number changes among individuals for most tissues profiled. However, our comparisons between cancer and paired normal tissues indicate that more that 90% of tumors had less than 2-fold increases or decreases for either 5S or 45S rDNA. Similarly, most 1q42 amplification corresponded to duplications of the segment with less than 15% of the cases corresponding to higher increases (ploidy ≥ 2.5). The naturally occurring “normal” variation also indicates that excess 45S rDNA CN does not limit cell viability, and argues against a model in which lower 45S rDNA CN facilitates tumorigenesis. There are plenty of healthy individuals with very small rDNA CN. What drives this natural variation remains to be established but one interpretation we previously advanced is that the 5S/45S ratio is constrained due to the stoichiometric demands [3]. How can rDNA copy number changes in cancer lineages be reconciled with large naturally occurring inter-individual variation in absolute rDNA copy number? One clue comes from the observation that somatic changes of genetic context in cancers as well as changes in proliferation rates and nucleolar activity influence rDNA CN. For instance, our analyses indicated that mutation in *TP53* is among the strongest determinants of coupled 5S gain and 45S loss in cancer lineages. Hence, the relative gain or loss of rDNA units is partially determined by somatic changes in genetic context as well as changes in nucleolar activity and proliferation occurring within individuals. However, these somatic alterations are relatively minor when compared to genetic differences between individuals. The data raise the possibility that rDNA CN has a polygenic basis with inter-individual variation in rDNA array copy number strongly determined by genetic background, with increases and decreases within cancers reflecting rDNA adaptations to accelerate proliferation rates in tumor cells.

All aspects of rapid cell proliferation are dependent on efficient ribosomal biogenesis to sustain protein synthesis. Ribosomal RNA molecules are necessary structural components of eukaryotic ribosomes and high transcription rates from several rDNA units in the multicopy rDNA array are necessary to provide sufficient rRNAs for ribosome biogenesis. Indeed, up-regulation of ribosomal genes in cancer has been documented and is presumed to reflect the increased translational demand in rapidly proliferating cells [40, 57, 58]. Hence, the amplification of the 5S rDNA array in cancer is presumably selected by an increased demand for 5S rRNA molecules. It is also conceivable that lowered 45S rDNA CN could be adaptive and positively selected in cancer. This is because excess of rDNA copies has been suggested to promote global genome stability [35], and epigenetic regulation of the 45S rDNA could compensate for the loss of 45S rDNA alleles. If this is the case, the shorter 45S rDNA array in cancer could contribute to increased tumor evolvability through the promotion of genome instability. In this regard, drugs that target the rDNA to induce loss might further fuel the adaptive ability of cancer lineages. On the other hand, 45S rDNA loss might be a byproduct of replication stress emerging from the challenge of maintaining fast replication rates and high transcription rates in the 45S rDNA in rapid proliferating cells. Replication stress might be particularly salient in cancers lineages undergoing rapid cell cycle at the limit of their replication stress capacity. Indeed, transcription-replication conflict is common in eukaryotic genomes [59]. If cancer cells are indeed at the lower limit of frailty to balance high rRNA transcription with rDNA replication and repair capacity, drugs that target rDNA array copy number to induce further loss could be particularly effective.

Collectively, our observations about natural rDNA variation among genotypes and in cancer lineages suggest that gains or losses of rDNA units in cancer relative to normal adjacent tissue are more relevant in tumors than absolute rDNA copy number. The gain of 5S copies is expected to enhance ribosomal and nucleolar function in cancers whereas 45S copy number loss is a byproduct of replication-transcription conflict in rapidly proliferating cells. Interestingly, 45S rDNA loci are epigenetically regulated [60–62], with only a fraction of the alleles in an array being expressed at any time. Uncoupling 5S and 45S rDNA CN in tumors raise the possibility that cancers might be particularly reliant on epigenetic mechanisms to achieve higher 45S rRNA expression in the face of 45S rDNA loss. In this regard, drugs targeting epigenetic states of the rDNA could be especially effective. Our observations raise the prospects of using 5S and 45S ribosomal DNA states as targets in new strategies for cancer therapy.

## Materials and methods

### Obtaining the expression data

The RNAseq reads count of genes for different cancer types were downloaded from the Genomic Data Commons (GDC) data portal (https://gdc-portal.nci.nih.gov/), and normalized separately for each cancer type by implementing the ‘TMM’ method from the edgeR library in R[63]. RPKMs were calculated following standard procedures. We only included genes represented by at least 10 reads in more than half of all individuals.

### Selecting reference rDNA and background sequences

We used the 43kb consensus sequence of the 45S rDNA (GenBank accession: U13369.1). This sequence was modified to ~16kb (combining nucleotides from 41021–42999 with 1–14000), so that the transcription start site, the three mature components (18S, 5.8S and 28S), as well as the 3’ external transcribed spacer regions were all included. The 5S rDNA sequences and flanking regions was identified in an earlier study [3] and directly used here. From the genome annotation of Ensembl GRCh37.82 [64], we extracted exons and introns on chromosome 1, 13, 14, 15, 21 and 22 (where the rDNA arrays are located). If exons from multiple isoforms overlapped each other, the largest exon was selected; on the other hand, if an intron overlaps with an exon from a different isoform, then the intron is removed. We then performed similar filters as earlier studies [3, 30] to obtain groups of single copy exons and introns according to the following criteria. First, exons and introns having BLAST hit (e <10–6) [65] with any region (except for itself) of any gene, or any annotated human repetitive sequences from Repbase21.10 [66], were removed. Next, exons and introns with length smaller than 300 bps or larger than 10 kbs were removed. Finally, the first and last 50 nts of each region were not included. As a result, 2,453 exons and 3,091 introns were identified on chromosome 1, and 2,825 exons and 3,237 introns were identified on chromosome 13, 14, 15, 21, 22.

### Obtaining rDNA array reads

The mapped genome sequencing data (BAM files) of cancer patients generated by the TCGA project were downloaded from the Legacy Archive website of the GDC data portal (https://gdc-portal.nci.nih.gov/legacy-archive/search/f). We obtained 113, 233, 50, 127, 44 and 154 tumor samples (24, 74, 32, 38, 35 and 22 tumor-adjacent pairs) for BLCA, LUAD, LUSC, STAD, KIRC and HNSC respectively; 9, 10 and 19 tumor-adjacent pairs for OV, BRCA and LIHC were also downloaded. We used two approaches to obtain reads mapped onto rDNA sequences. In the first approach, BAM files were converted back to raw FASTQ reads using bamUtil v1.0.13 (https://github.com/statgen/bamUtil), and then mapped *de novo* onto the rDNA consensus sequences with BWA v0.7.9a [67]. These *de novo* mapping and rDNA estimates are similar to our earlier implementations [3, 30]. This approach could be quite computation consuming for samples with high sequencing depth. Alternatively, we noticed that (i) nearly all of the rDNA reads are mapped onto the GL000220.1 scaffold (for 45S) as well as 1q42 (for 5S) [3, 30], and that (ii) most of the samples had been mapped onto hg19 chromosomes plus the unintegrated scaffolds. Hence, we then used Samtools v1.3.1[68] to directly slice BAMs files and extract reads that had been aligned onto scaffold GL000220.1, and the 1q42 region (chr1:226743523–231781906. That is, up to 2MBs flanking both sides of the annotated 5S array, or chr1: 228743523–228781906, were selected). After slicing, we aligned the subsets of reads onto the rDNA reference sequences. Note that multiple pseudo rRNA genes scatter across the genome. To account for the potential influence of these pseudo rRNAs for the first approach, we further mapped the rDNA reads onto human genome to calculate a correction ratio for each component, as suggested by earlier studies [3, 30]. However, it is not necessary to control for pseudo rRNAs in the slicing approach. We compared the results calculated from both approaches: only marginal differences were detected (S2C Fig). Hence, we adopted the slicing method throughout the study, because of its higher computational efficiency and comparable accuracy with *de novo* mapping.

### Estimation of background reads depth (BRD) for single copy exons and introns

From the downloaded BAM files, we calculated per base reads depth for the above groups of single copy exons and introns using the ‘depth’ command of Samtools. Note that cancer genomes suffer from large-scale structural variation, with frequent gain or loss of whole genes, partial segments as well as large-scale chromosomal duplications/losses. Therefore, we cannot assume that the selected background regions will necessarily maintain diploidy in cancer lineages. To account for the potential aneuploidy, we downloaded the gene-level copy number estimations produced as of January 28 2016 from the Broad FireBrowse portal. In this pipeline, genomic regions that undergo focal or arm-level amplification or deletion in tumors were identified by using the software GISTIC2 [43] from the human SNP array 6.0 datasets. One of the output files, ‘all_data_by_genes.txt’, contains gene-level copy number values (denoted as “C”) for each tumor sample. Here C = 0 means normal diploid, and 2 + C indicates the actual ploidy of the gene. We noticed a ranging ploidy for genes in tumor, supporting a need for correction (S1 Fig). Ideally the ploidy of a gene corresponds with its sequencing depth. Encouragingly, we did observe strong positive correlations in tumors (S2A Fig). Therefore, we used a ratio, R = (2 + C)/2 to represent the fold change of gene copy in tumor relative to normal. We corrected the per-base coverage depth of selected exons and introns by the corresponding gene’s R-value in each tumor sample. The mean value of the adjusted depths was then used as BRD in tumors. On the other hand, BRD of adjacent or blood was computed as before without correction for ploidy [3, 30]. The correction process leads to improved rDNA CN estimates in tumors.

### Calculation of rDNA copy number (CN)

After obtaining reads mapped onto the rDNA references, we calculated the per-base depth for each component. The depths of 5.8S and 5S were averaged across all sites, a robust approach in view of the nearly identical depth estimates along these components. On the other hand, the two larger components, especially 28S, displayed considerable variability in depths across sites, which reflect underlying sequence polymorphism. To account for such heterogeneity, we considered two approaches. In the first approach, the mean depth is calculated as the average of the selected region of 18S (901–1871) or whole 28S sequence respectively. The reason to use the selected region to represent 18S is that the first ~600 bps of 18S has a homologous segment on chromosome 21 [30]. Since the slicing approach only recovered reads mapped onto scaffold GL000220.1 (for 45S), the depth of 18S is underestimated for the first ~600 bps. To remove the potential inaccuracy, we therefore only considered the last 971 bps of the 18S. Alternatively, we examined a 150bp sliding-window in each component. For each 1 bp step we calculated the coefficient of variation (CV) of average depth across samples for the 150 nucleotides. The window with the lowest mean CV across LUAD adjacent samples were selected (18S:1145–1294, 28S:1522–1671). Moreover, we found that LUAD tumors have similar CV along 18S and 28S as adjacent, and that the selected windows also tend to have almost the least CV in LUAD tumors (Fig 1A), supporting the robustness of the selection. The same windows were applied for all other cancer types. The segment/window-based method overall yielded better estimates for 18S and 28S (Fig 1B), and qualitatively very similar conclusions (S4 Fig and S2 Table), compared to the full-length method. Finally, to further obtain rDNA copy numbers, we divided the mean depth of 5S by the mean BRD of selected exons and introns from chromosome 1, whereas mean depth of 45S components were divided by BRD calculated from exons in chromosome 13, 14, 15, 21, 22, with corrections for aneuploidy in both cases. We also estimated CN for the overall 45S array by using the averaged depth across 18S, 5.8S and 28S components, with the slid windows applied for 18S and 28S.

### Identifying the plate ID effect and intra-plate variation

Plate ID (corresponding to sequencing center) of TCGA exome/genome sequencing data has been shown to affect CN estimates in previous studies [44, 69]. Hence, we carefully inspected our rDNA CN estimates to identify such effects. Specifically, since a number of samples were separately processed in different batches, we selected pairs of batches that shared at least 6 samples, and used paired Wilcoxon rank sum tests to identify potential differences between pairs from different batches. Ideally, shared samples should have similar CN estimates in each batch. However, as observed before, we did also observe significant differences for some comparisons (S2B Fig and S1 Table). To account for such influence, we adopted the approach from [44]. Briefly, for each cancer type, a linear regression model was applied with tissue type and batch as independent variables, and logarithmic rDNA CN as the dependent variable. All 4 components, as well as the 45S array estimated above were corrected separately for each cancer type.

### Associating rDNA CN with somatic copy number alterations and non-silent mutations

To associate genome wide SCNAs with rDNA CN, we used significant local level amplification and deletion events identified in the FireBrowse portal. The extent of the SCNA is measured as “ploidy − 2” for amplification, and “2 − ploidy” for deletion. Tumors with a local ploidy change value larger than 0.1 (for amplifications) or smaller than -0.1 (for deletions) were identified as containing the SCNA. For each SCNA, we used a linear regression model to test the association between the SCNA event and the change in rDNA CN. Similarly, to associate somatic mutations with rDNA CN, we used “.maf” input file of the mutation analysis pipelines in the FireBrowse portal. We only included genes with non-silent somatic mutations in at least 10 patients. We used a Wilcoxon rank sum test to compare rDNA CN with in tumor lineages with and without the focal mutation.

### Calculating proliferation indexes (PRI), cRPG expression, and nucleolar expression

We used two sets of independently identified genes to calculate PRI. The first set with 793 genes (or the ‘YW’ set) was obtained from Yedael Y. Waldman et al. [55], who calculated PRI from genes significantly positively correlated (Spearman’s **ρ** > 0, *P* < 0.01) with cell proliferation across 60 cancer cell lines. The second set of 350 genes (or the ‘RS’ set) was compiled by Rickard Sandberg et al. [56], who proposed the use of PRIs based on cell proliferation estimates obtained in non-cancerous cell lineages. Here we used the median expression of selected subset of genes to represent PRI. Interestingly, only 74 genes are shared between the two sets, yet both sets yielded highly correlated PRIs (S6A Fig). To answer which cancers have significantly different PRI relative to adjacent tissue, the median FC of PRI genes between adjacent and tumor was calculated across patients. A minimum of 5 patients with paired adjacent and tumor RNAseq data for each cancer type were required. cRPG genes are defined as the protein components of the large and small ribosomal subunits (GO:0022625 and GO:0022627; 100 genes). Nucleolar activity is defined as the median expression of the 799 genes belonging to the cellular component “nucleolus” (GO:0005730). The lists of cRPGs and nucleolar protein genes are in S6 Table.

### Availability of data and materials

The RNAseq processed data were downloaded from the GDC data portal (https://gdc-portal.nci.nih.gov/). The mapped genome sequencing data, under dbGaP accession phs000178.v9.p8, were downloaded from the Legacy Archive website of the GDC data portal (https://gdc-portal.nci.nih.gov/legacy-archive/search/f). The copy number variation and somatic mutation data were downloaded the FireBrowse portal (http://firebrowse.org/). All the data of cancer patients were generated or processed by the TCGA project (https://cancergenome.nih.gov/) and (http://www.ensembl.org/index.html). The two sets of genes used in the computation of the proliferation indexes were identified previously [55, 56].

## Supporting information

S1 Fig The distribution of ploidy for genes across cancers. Genes on (A) chromosome 1 and (B) five others (13, 14, 15, 21, 22) were used. Each gene from each tumor sample was regarded as an event.
(PDF)

S2 Fig Estimating rDNA copy number in cancers. (A) Sequencing depth in exons (Spearman’s **ρ** = 0.71, *P* < 2.2e-16) and introns (**ρ** = 0.69, *P* < 2.2e-16) are strongly correlated with a gene’s ploidy in tumor. A LUAD tumor sample (TCGA-91-6847-01A-11D-1945-08) was randomly selected for this display. (B) Identical samples processed from batch 1600 had higher 28S copies than those of batch 1598 (Paired Wilcoxon rank sum test, *P* = 0.002). (C) Copy number (CN) estimates for all 4 components using two approaches (*de novo* mapping of raw reads or by “slicing” from pre-processed BAM files) are nearly identical across 100 randomly selected LUAD samples.
(PDF)

S3 Fig Pairwise Pearson correlation coefficients between rDNA components. Error bars show the 95% confidence intervals.
(PDF)

S4 Fig Variable manifestation of concerted copy number variation (cCNV) across tissues. This figure is similar to Fig 3, except that the 901–1871 bps of 18S and the full length of 28S were used when calculating the depths and CN of the 45S.
(PDF)

S5 Fig *MDM2* is significantly amplified in LUAD, LUSC, BLCA and STAD, but not in HNSC and KIRC. (PDF)

S6 Fig The two independently defined PRI gene sets yield consistent results. (A) PRIs calculated from the YW and RS gene sets are strongly correlated. (B) All cancer types have increased PRI except KICH. Seventeen cancer types with RNA-seq data in ≥ 5 tumor-adjacent pairs were shown, with sample sizes in brackets. Yellow and grey indicate significant up-regulation (Wilcoxon rank sum test *P* < 0.01) and not significant in tumors compared with paired adjacent controls, respectively. (C) The Spearman’s correlation coefficients of PRI with nucleolar genes are significantly higher than that with cRPGs for the 17 cancer types (*P* from paired Wilcoxon rank sum test). RS set was used in B and C. COAD, colon adenocarcinoma; KIRP, kidney renal papillary cell carcinoma; THCA, thyroid carcinoma; READ, rectum adenocarcinoma; KICH, kidney chromophobe; PRAD, prostate adenocarcinoma; CHOL, cholangiocarcinoma; UCEC, uterine corpus endometrial carcinoma; ESCA, esophageal carcinoma. Other abbreviations are as in Table 1.
(PDF)

S7 Fig Higher proliferation rate is linked to more aggressive tumors. Example using LUAD data show that patients with higher PRI had (A, B) worse survival (comparing the last with the first 25% patients, logrank test, *P* < 0.006, Hazards ratio > 2.35), as well as (C, D) more severe tumor stage (ANOVA, *P* < 0.0075). The YW gene set is used for A and C; while the RS gene set is used for B and D.
(PDF)

S8 Fig Venn diagrams showing the overlap between gene sets classified as cRPGs or nucleolar and gene sets used to compute PRI. Note that the gene-set used to calculate PRI is mostly distinct from the gene-set used to calculate nucleolar activity.
(PDF)

S9 Fig Associations between tumor proliferation (RS set) and rDNA copy number. (A) PRI is significant negatively correlated with 45S and positively with the 5S / 45S ratio whereas it is not significant with 5S in tumors. (B) Consistent results were observed when associating tumor vs. adjacent normal fold change of PRI with that of 5S, 45S or their ratio for 31 patients. RS set genes and LUAD samples are used.
(PDF)

S10 Fig Associations between tumor proliferation (YW set) and rDNA copy number. Spearman correlations between relative fold change of proliferation in tumor relative to its adjacent, and the fold change of 5S, 45S or their ratio for same patients. LUAD samples are used.
(PDF)

S1 Table Sequencing batch (plate ID) effects on rDNA CN estimation. Samples sequenced on two separate plates (plate 1 and plate 2) were identified. These were selected and their CN compared with a Wilcox rank sum test.
(XLSX)

S2 Table Tumor vs. adjacent normal 45S CN loss is robustly detected when copy number estimates were obtained with the full-length method and with the segment method. (XLSX)

S3 Table Association between rDNA CN with somatic copy number alternations (SCNAs). (XLSX)

S4 Table Association between rDNA CN with somatic mutations. Each cancer type was analyzed separately.
(XLSX)

S5 Table Association between rDNA CN with somatic mutations. All available cancers were pooled.
(XLSX)

S6 Table The lists of cytoplasmic ribosomal protein genes and nucleolar protein genes. (XLSX)

S7 Table Association between rDNA CN and proliferation index (PRI) in tumors. (XLSX)

S8 Table Pan-cancer association of rDNA CN with somatic mutations and PRI. (XLSX)

## References

[1] McConkey EH, Hopkins JW. The Relationship of the Nucleolus to the Synthesis of Ribosomal Rna in Hela Cells. Proc Natl Acad Sci U S A. 1964;51:1197–204. . 14215644 10.1073/pnas.51.6.1197 PMC300235

[2] Richard GF, Kerrest A, Dujon B. Comparative genomics and molecular dynamics of DNA repeats in eukaryotes. Microbiol Mol Biol Rev. 2008;72(4):686–727. doi: 10.1128/MMBR.00011-08 . 19052325 10.1128/MMBR.00011-08 PMC2593564

[3] Gibbons JG, Branco AT, Godinho SA, Yu S, Lemos B. Concerted copy number variation balances ribosomal DNA dosage in human and mouse genomes. Proc Natl Acad Sci U S A. 2015;112(8):2485–90. doi: 10.1073/pnas.1416878112 . 25583482 10.1073/pnas.1416878112 PMC4345604

[4] Stults DM, Killen MW, Williamson EP, Hourigan JS, Vargas HD, Arnold SM, et al Human rRNA gene clusters are recombinational hotspots in cancer. Cancer Res. 2009;69(23):9096–104. doi: 10.1158/0008-5472.CAN-09-2680 . 19920195 10.1158/0008-5472.CAN-09-2680

[5] Stults DM, Killen MW, Pierce HH, Pierce AJ. Genomic architecture and inheritance of human ribosomal RNA gene clusters. Genome Res. 2008;18(1):13–8. doi: 10.1101/gr.6858507 . 18025267 10.1101/gr.6858507 PMC2134781

[6] Schwarzacher HG, Wachtler F. The nucleolus. Anatomy and embryology. 1993;188(6):515–36. Epub 1993/12/01. . 8129175 10.1007/BF00187008

[7] Klein J, Grummt I. Cell cycle-dependent regulation of RNA polymerase I transcription: the nucleolar transcription factor UBF is inactive in mitosis and early G1. Proc Natl Acad Sci U S A. 1999;96(11):6096–101. . 10339547 10.1073/pnas.96.11.6096 PMC26841

[8] Kief DR, Warner JR. Coordinate control of syntheses of ribosomal ribonucleic acid and ribosomal proteins during nutritional shift-up in Saccharomyces cerevisiae. Mol Cell Biol. 1981;1(11):1007–15. . 7050661 10.1128/mcb.1.11.1007-1015.1981 PMC369722

[9] Grummt I. Regulation of mammalian ribosomal gene transcription by RNA polymerase I. Prog Nucleic Acid Res Mol Biol. 1999;62:109–54. . 9932453 10.1016/s0079-6603(08)60506-1

[10] Hogues H, Lavoie H, Sellam A, Mangos M, Roemer T, Purisima E, et al Transcription factor substitution during the evolution of fungal ribosome regulation. Mol Cell. 2008;29(5):552–62. doi: 10.1016/j.molcel.2008.02.006 . 18342603 10.1016/j.molcel.2008.02.006 PMC3838363

[11] Stuart JM, Segal E, Koller D, Kim SK. A gene-coexpression network for global discovery of conserved genetic modules. Science. 2003;302(5643):249–55. doi: 10.1126/science.1087447 . 12934013 10.1126/science.1087447

[12] Tanay A, Regev A, Shamir R. Conservation and evolvability in regulatory networks: the evolution of ribosomal regulation in yeast. Proc Natl Acad Sci U S A. 2005;102(20):7203–8. doi: 10.1073/pnas.0502521102 . 15883364 10.1073/pnas.0502521102 PMC1091753

[13] Thorrez L, Van Deun K, Tranchevent LC, Van Lommel L, Engelen K, Marchal K, et al Using ribosomal protein genes as reference: a tale of caution. PLoS One. 2008;3(3):e1854 doi: 10.1371/journal.pone.0001854 . 18365009 10.1371/journal.pone.0001854 PMC2267211

[14] Wapinski I, Pfiffner J, French C, Socha A, Thompson DA, Regev A. Gene duplication and the evolution of ribosomal protein gene regulation in yeast. Proc Natl Acad Sci U S A. 2010;107(12):5505–10. doi: 10.1073/pnas.0911905107 . 20212107 10.1073/pnas.0911905107 PMC2851827

[15] Guimaraes JC, Zavolan M. Patterns of ribosomal protein expression specify normal and malignant human cells. Genome Biol. 2016;17(1):236 doi: 10.1186/s13059-016-1104-z . 27884178 10.1186/s13059-016-1104-z PMC5123215

[16] Juli G, Gismondi A, Monteleone V, Caldarola S, Iadevaia V, Aspesi A, et al Depletion of ribosomal protein S19 causes a reduction of rRNA synthesis. Sci Rep. 2016;6:35026 doi: 10.1038/srep35026 . 27734913 10.1038/srep35026 PMC5062126

[17] Ramaswamy P, Woodson SA. Global stabilization of rRNA structure by ribosomal proteins S4, S17, and S20. J Mol Biol. 2009;392(3):666–77. doi: 10.1016/j.jmb.2009.07.032 . 19616559 10.1016/j.jmb.2009.07.032 PMC2763185

[18] Ziemiecki A, Muller RG, Fu XC, Hynes NE, Kozma S. Oncogenic activation of the human trk proto-oncogene by recombination with the ribosomal large subunit protein L7a. EMBO J. 1990;9(1):191–6. . 2403926 10.1002/j.1460-2075.1990.tb08095.x PMC551645

[19] Deisenroth C, Zhang Y. Ribosome biogenesis surveillance: probing the ribosomal protein-Mdm2-p53 pathway. Oncogene. 2010;29(30):4253–60. doi: 10.1038/onc.2010.189 . 20498634 10.1038/onc.2010.189

[20] Boulon S, Westman BJ, Hutten S, Boisvert FM, Lamond AI. The nucleolus under stress. Mol Cell. 2010;40(2):216–27. doi: 10.1016/j.molcel.2010.09.024 . 20965417 10.1016/j.molcel.2010.09.024 PMC2987465

[21] James A, Wang Y, Raje H, Rosby R, DiMario P. Nucleolar stress with and without p53. Nucleus. 2014;5(5):402–26. doi: 10.4161/nucl.32235 25482194 10.4161/nucl.32235 PMC4164484

[22] Ruggero D. Revisiting the nucleolus: from marker to dynamic integrator of cancer signaling. Sci Signal. 2012;5(241):pe38 doi: 10.1126/scisignal.2003477 . 22969157 10.1126/scisignal.2003477 PMC4390046

[23] Hannan KM, Hannan RD, Smith SD, Jefferson LS, Lun M, Rothblum LI. Rb and p130 regulate RNA polymerase I transcription: Rb disrupts the interaction between UBF and SL-1. Oncogene. 2000;19(43):4988–99. doi: 10.1038/sj.onc.1203875 . 11042686 10.1038/sj.onc.1203875

[24] Zhai W, Comai L. Repression of RNA polymerase I transcription by the tumor suppressor p53. Mol Cell Biol. 2000;20(16):5930–8. . 10913176 10.1128/mcb.20.16.5930-5938.2000 PMC86070

[25] Hannan KM, Kennedy BK, Cavanaugh AH, Hannan RD, Hirschler-Laszkiewicz I, Jefferson LS, et al RNA polymerase I transcription in confluent cells: Rb downregulates rDNA transcription during confluence-induced cell cycle arrest. Oncogene. 2000;19(31):3487–97. doi: 10.1038/sj.onc.1203690 . 10918607 10.1038/sj.onc.1203690

[26] Menssen A, Hermeking H. Characterization of the c-MYC-regulated transcriptome by SAGE: identification and analysis of c-MYC target genes. Proc Natl Acad Sci U S A. 2002;99(9):6274–9. doi: 10.1073/pnas.082005599 . 11983916 10.1073/pnas.082005599 PMC122939

[27] Kim S, Li Q, Dang CV, Lee LA. Induction of ribosomal genes and hepatocyte hypertrophy by adenovirus-mediated expression of c-Myc in vivo. Proc Natl Acad Sci U S A. 2000;97(21):11198–202. doi: 10.1073/pnas.200372597 . 11005843 10.1073/pnas.200372597 PMC17177

[28] Boon K, Caron HN, van Asperen R, Valentijn L, Hermus MC, van Sluis P, et al N-myc enhances the expression of a large set of genes functioning in ribosome biogenesis and protein synthesis. EMBO J. 2001;20(6):1383–93. doi: 10.1093/emboj/20.6.1383 . 11250904 10.1093/emboj/20.6.1383 PMC145518

[29] Eagle SH, Crease TJ. Copy number variation of ribosomal DNA and Pokey transposons in natural populations of Daphnia. Mob DNA. 2012;3(1):4 doi: 10.1186/1759-8753-3-4 . 22390386 10.1186/1759-8753-3-4 PMC3315735

[30] Gibbons JG, Branco AT, Yu S, Lemos B. Ribosomal DNA copy number is coupled with gene expression variation and mitochondrial abundance in humans. Nat Commun. 2014;5:4850 doi: 10.1038/ncomms5850 . 25209200 10.1038/ncomms5850

[31] Lyckegaard EM, Clark AG. Evolution of ribosomal RNA gene copy number on the sex chromosomes of Drosophila melanogaster. Mol Biol Evol. 1991;8(4):458–74. . 1921706 10.1093/oxfordjournals.molbev.a040664

[32] Warner JR. The economics of ribosome biosynthesis in yeast. Trends Biochem Sci. 1999;24(11):437–40. . 10542411 10.1016/s0968-0004(99)01460-7

[33] Hontz RD, French SL, Oakes ML, Tongaonkar P, Nomura M, Beyer AL, et al Transcription of multiple yeast ribosomal DNA genes requires targeting of UAF to the promoter by Uaf30. Mol Cell Biol. 2008;28(21):6709–19. doi: 10.1128/MCB.00703-08 . 18765638 10.1128/MCB.00703-08 PMC2573240

[34] Nemeth A, Langst G. Chromatin organization of active ribosomal RNA genes. Epigenetics. 2008;3(5):243–5. . 18948749 10.4161/epi.3.5.6913

[35] Ide S, Miyazaki T, Maki H, Kobayashi T. Abundance of ribosomal RNA gene copies maintains genome integrity. Science. 2010;327(5966):693–6. doi: 10.1126/science.1179044 . 20133573 10.1126/science.1179044

[36] Paredes S, Branco AT, Hartl DL, Maggert KA, Lemos B. Ribosomal DNA deletions modulate genome-wide gene expression: "rDNA-sensitive" genes and natural variation. PLoS Genet. 2011;7(4):e1001376 doi: 10.1371/journal.pgen.1001376 . 21533076 10.1371/journal.pgen.1001376 PMC3080856

[37] Derenzini M, Trere D, Pession A, Montanaro L, Sirri V, Ochs RL. Nucleolar function and size in cancer cells. Am J Pathol. 1998;152(5):1291–7. . 9588897 PMC1858570

[38] Derenzini M, Trere D, Pession A, Govoni M, Sirri V, Chieco P. Nucleolar size indicates the rapidity of cell proliferation in cancer tissues. J Pathol. 2000;191(2):181–6. doi: 10.1002/(SICI)1096-9896(200006)191:2<181::AID-PATH607>3.0.CO;2-V . 10861579 10.1002/(SICI)1096-9896(200006)191:2<181::AID-PATH607>3.0.CO;2-V

[39] Smetana K. Structural features of nucleoli in blood, leukemic, lymphoma and myeloma cells. Eur J Histochem. 2002;46(2):125–32. . 12152790 10.4081/1661

[40] Montanaro L, Trere D, Derenzini M. Nucleolus, ribosomes, and cancer. Am J Pathol. 2008;173(2):301–10. doi: 10.2353/ajpath.2008.070752 . 18583314 10.2353/ajpath.2008.070752 PMC2475768

[41] Storchova Z, Pellman D. From polyploidy to aneuploidy, genome instability and cancer. Nat Rev Mol Cell Biol. 2004;5(1):45–54. doi: 10.1038/nrm1276 . 14708009 10.1038/nrm1276

[42] Negrini S, Gorgoulis VG, Halazonetis TD. Genomic instability—an evolving hallmark of cancer. Nat Rev Mol Cell Biol. 2010;11(3):220–8. doi: 10.1038/nrm2858 . 20177397 10.1038/nrm2858

[43] Mermel CH, Schumacher SE, Hill B, Meyerson ML, Beroukhim R, Getz G. GISTIC2.0 facilitates sensitive and confident localization of the targets of focal somatic copy-number alteration in human cancers. Genome Biol. 2011;12(4):R41 doi: 10.1186/gb-2011-12-4-r41 . 21527027 10.1186/gb-2011-12-4-r41 PMC3218867

[44] Reznik E, Miller ML, Senbabaoglu Y, Riaz N, Sarungbam J, Tickoo SK, et al Mitochondrial DNA copy number variation across human cancers. Elife. 2016;5 doi: 10.7554/eLife.10769 . 26901439 10.7554/eLife.10769 PMC4775221

[45] Caburet S, Conti C, Schurra C, Lebofsky R, Edelstein SJ, Bensimon A. Human ribosomal RNA gene arrays display a broad range of palindromic structures. Genome Res. 2005;15(8):1079–85. doi: 10.1101/gr.3970105 . 16024823 10.1101/gr.3970105 PMC1182220

[46] Zafiropoulos A, Tsentelierou E, Linardakis M, Kafatos A, Spandidos DA. Preferential loss of 5S and 28S rDNA genes in human adipose tissue during ageing. Int J Biochem Cell Biol. 2005;37(2):409–15. doi: 10.1016/j.biocel.2004.07.007 . 15474985 10.1016/j.biocel.2004.07.007

[47] Claycomb JM, Orr-Weaver TL. Developmental gene amplification: insights into DNA replication and gene expression. Trends Genet. 2005;21(3):149–62. doi: 10.1016/j.tig.2005.01.009 . 15734574 10.1016/j.tig.2005.01.009

[48] Loeb LA, Loeb KR, Anderson JP. Multiple mutations and cancer. Proc Natl Acad Sci U S A. 2003;100(3):776–81. doi: 10.1073/pnas.0334858100 . 12552134 10.1073/pnas.0334858100 PMC298677

[49] Pino MS, Chung DC. The chromosomal instability pathway in colon cancer. Gastroenterology. 2010;138(6):2059–72. doi: 10.1053/j.gastro.2009.12.065 . 20420946 10.1053/j.gastro.2009.12.065 PMC4243705

[50] Sloan KE, Bohnsack MT, Watkins NJ. The 5S RNP couples p53 homeostasis to ribosome biogenesis and nucleolar stress. Cell Rep. 2013;5(1):237–47. doi: 10.1016/j.celrep.2013.08.049 . 24120868 10.1016/j.celrep.2013.08.049 PMC3808153

[51] Berthon P, Valeri A, Cohen-Akenine A, Drelon E, Paiss T, Wohr G, et al Predisposing gene for early-onset prostate cancer, localized on chromosome 1q42.2–43. American journal of human genetics. 1998;62(6):1416–24. . 9585607 10.1086/301879 PMC1377158

[52] Zack TI, Schumacher SE, Carter SL, Cherniack AD, Saksena G, Tabak B, et al Pan-cancer patterns of somatic copy number alteration. Nat Genet. 2013;45(10):1134–40. doi: 10.1038/ng.2760 . 24071852 10.1038/ng.2760 PMC3966983

[53] Olivier M, Hollstein M, Hainaut P. TP53 mutations in human cancers: origins, consequences, and clinical use. Cold Spring Harb Perspect Biol. 2010;2(1):a001008 doi: 10.1101/cshperspect.a001008 . 20182602 10.1101/cshperspect.a001008 PMC2827900

[54] Ruggero D, Pandolfi PP. Does the ribosome translate cancer? Nat Rev Cancer. 2003;3(3):179–92. doi: 10.1038/nrc1015 . 12612653 10.1038/nrc1015

[55] Waldman YY, Geiger T, Ruppin E. A genome-wide systematic analysis reveals different and predictive proliferation expression signatures of cancerous vs. non-cancerous cells. PLoS Genet. 2013;9(9):e1003806 doi: 10.1371/journal.pgen.1003806 . 24068970 10.1371/journal.pgen.1003806 PMC3778010

[56] Sandberg R, Neilson JR, Sarma A, Sharp PA, Burge CB. Proliferating cells express mRNAs with shortened 3' untranslated regions and fewer microRNA target sites. Science. 2008;320(5883):1643–7. doi: 10.1126/science.1155390 . 18566288 10.1126/science.1155390 PMC2587246

[57] Truitt ML, Ruggero D. New frontiers in translational control of the cancer genome. Nat Rev Cancer. 2016;16(5):288–304. doi: 10.1038/nrc.2016.27 . 27112207 10.1038/nrc.2016.27 PMC5491099

[58] Pogue-Geile K, Geiser JR, Shu M, Miller C, Wool IG, Meisler AI, et al Ribosomal protein genes are overexpressed in colorectal cancer: isolation of a cDNA clone encoding the human S3 ribosomal protein. Mol Cell Biol. 1991;11(8):3842–9. . 1712897 10.1128/mcb.11.8.3842-3849.1991 PMC361167

[59] Garcia-Muse T, Aguilera A. Transcription-replication conflicts: how they occur and how they are resolved. Nat Rev Mol Cell Biol. 2016;17(9):553–63. doi: 10.1038/nrm.2016.88 . 27435505 10.1038/nrm.2016.88

[60] Zentner GE, Saiakhova A, Manaenkov P, Adams MD, Scacheri PC. Integrative genomic analysis of human ribosomal DNA. Nucleic Acids Res. 2011;39(12):4949–60. doi: 10.1093/nar/gkq1326 . 21355038 10.1093/nar/gkq1326 PMC3130253

[61] Bird AP, Taggart MH, Gehring CA. Methylated and unmethylated ribosomal RNA genes in the mouse. J Mol Biol. 1981;152(1):1–17. . 6279862 10.1016/0022-2836(81)90092-9

[62] Carvalho A, Polanco C, Lima-Brito J, Guedes-Pinto H. Differential rRNA Genes Expression in Hexaploid Wheat Related to NOR Methylation. Plant Molecular Biology Reporter. 2010;28(3):403–12. doi: 10.1007/s11105-009-0165-5

[63] Robinson MD, McCarthy DJ, Smyth GK. edgeR: a Bioconductor package for differential expression analysis of digital gene expression data. Bioinformatics. 2010;26(1):139–40. doi: 10.1093/bioinformatics/btp616 . 19910308 10.1093/bioinformatics/btp616 PMC2796818

[64] Yates A, Akanni W, Amode MR, Barrell D, Billis K, Carvalho-Silva D, et al Ensembl 2016. Nucleic Acids Res. 2016;44(D1):D710–6. doi: 10.1093/nar/gkv1157 . 26687719 10.1093/nar/gkv1157 PMC4702834

[65] Altschul SF, Madden TL, Schaffer AA, Zhang J, Zhang Z, Miller W, et al Gapped BLAST and PSI-BLAST: a new generation of protein database search programs. Nucleic Acids Res. 1997;25(17):3389–402. . 9254694 10.1093/nar/25.17.3389 PMC146917

[66] Bao W, Kojima KK, Kohany O. Repbase Update, a database of repetitive elements in eukaryotic genomes. Mob DNA. 2015;6:11 doi: 10.1186/s13100-015-0041-9 . 26045719 10.1186/s13100-015-0041-9 PMC4455052

[67] Li H, Durbin R. Fast and accurate short read alignment with Burrows-Wheeler transform. Bioinformatics. 2009;25(14):1754–60. doi: 10.1093/bioinformatics/btp324 . 19451168 10.1093/bioinformatics/btp324 PMC2705234

[68] Li H, Handsaker B, Wysoker A, Fennell T, Ruan J, Homer N, et al The Sequence Alignment/Map format and SAMtools. Bioinformatics. 2009;25(16):2078–9. doi: 10.1093/bioinformatics/btp352 . 19505943 10.1093/bioinformatics/btp352 PMC2723002

[69] Ju YS, Alexandrov LB, Gerstung M, Martincorena I, Nik-Zainal S, Ramakrishna M, et al Origins and functional consequences of somatic mitochondrial DNA mutations in human cancer. Elife. 2014;3 doi: 10.7554/eLife.02935 . 25271376 10.7554/eLife.02935 PMC4371858

