# Supplementary figures and images for: Ribosomal DNA copy number amplification and loss in human cancers is linked to tumor genetic context, nucleolus activity, and proliferation
Source: PLoS Genet. 2017 Sep 7;13(9):e1006994. doi: 10.1371/journal.pgen.1006994 (PMC5605086; doi:10.1371/journal.pgen.1006994)

**A**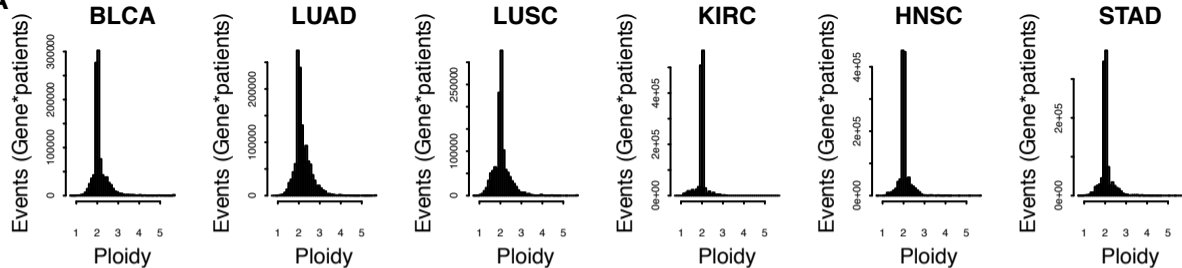**B**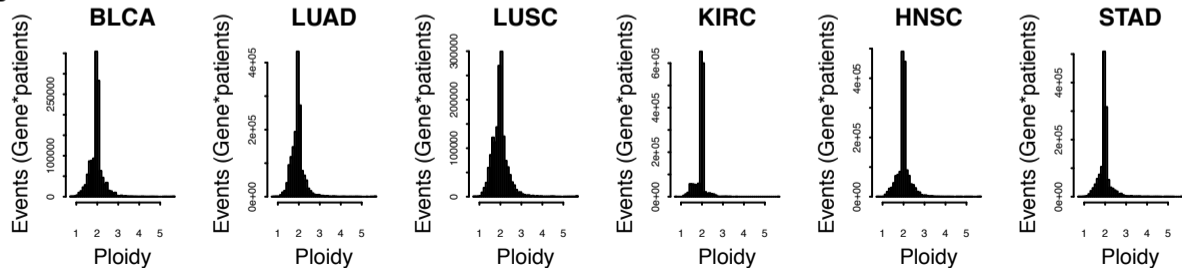

Supplement: S1 Fig — Genes on (A) chromosome 1 and (B) five others (13, 14, 15, 21, 22) were used. Each gene from each tumor sample was regarded as an event. (PDF) [file pgen.1006994.s001.pdf]

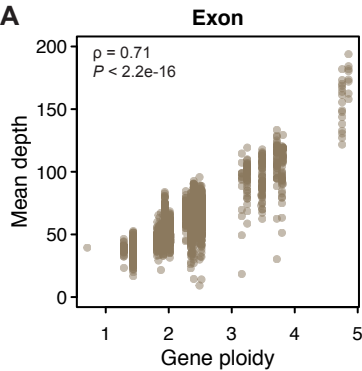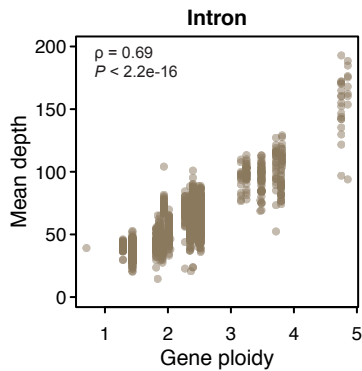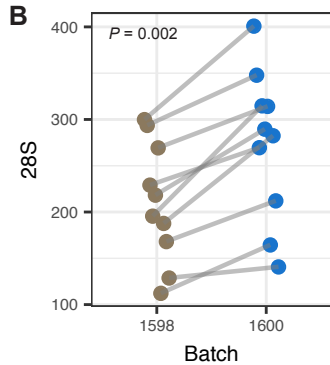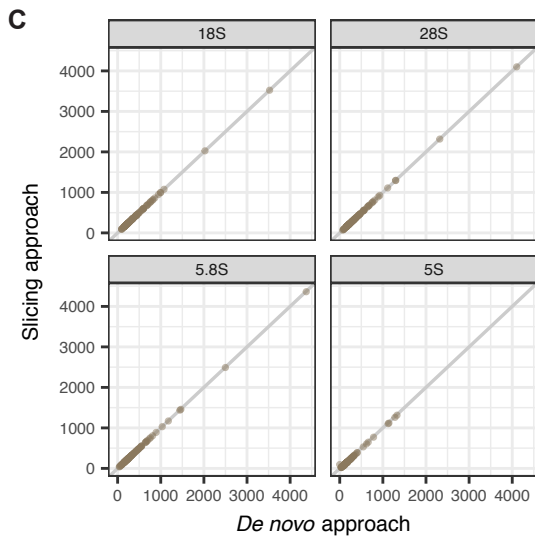

Supplement: S2 Fig — (A) Sequencing depth in exons (Spearman’s ρ = 0.71, P < 2.2e-16) and introns (ρ = 0.69, P < 2.2e-16) are strongly correlated with a gene’s ploidy in tumor. A LUAD tumor sample (TCGA-91-6847-01A-11D-1945-08) was randomly selected for this display. (B) Identical samples processed from batch 1600 had higher 28S copies than those of batch 1598 (Paired Wilcoxon rank sum test, P = 0.002). (C) Copy number (CN) estimates for all 4 components using two approaches (de novo mapping of raw reads or by “slicing” from pre-processed BAM files) are nearly identical across 100 randomly selected LUAD samples. (PDF) [file pgen.1006994.s002.pdf]

Correlation Coefficients

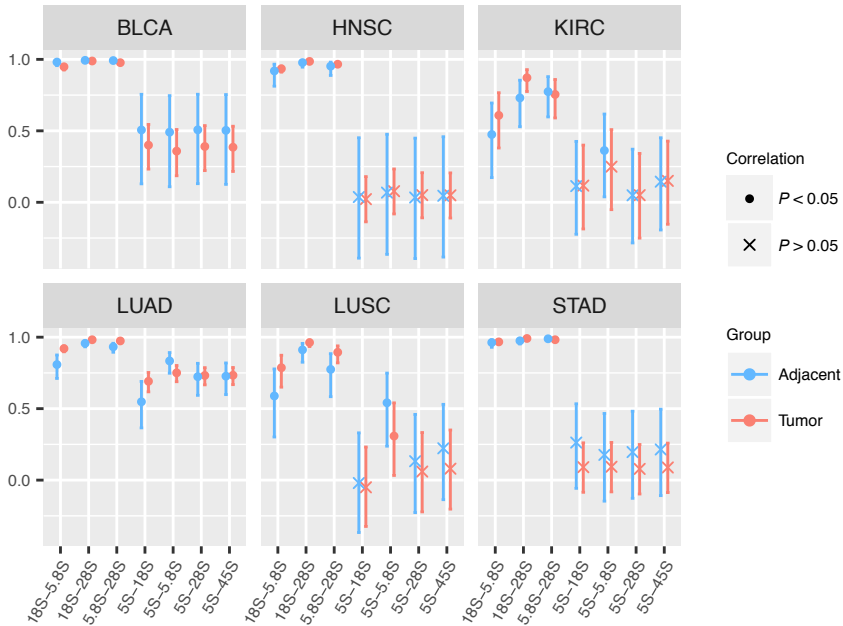

Supplement: S3 Fig — Error bars show the 95% confidence intervals. (PDF) [file pgen.1006994.s003.pdf]

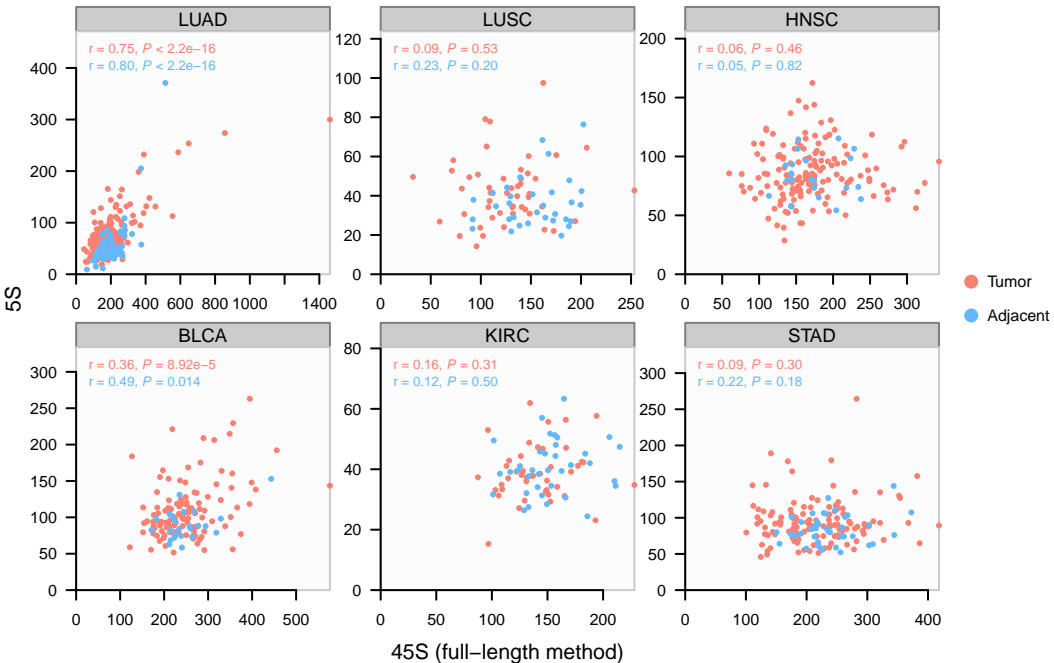

Supplement: S4 Fig — This figure is similar to Fig 3, except that the 901–1871 bps of 18S and the full length of 28S were used when calculating the depths and CN of the 45S. (PDF) [file pgen.1006994.s004.pdf]

MDM2 ploidy

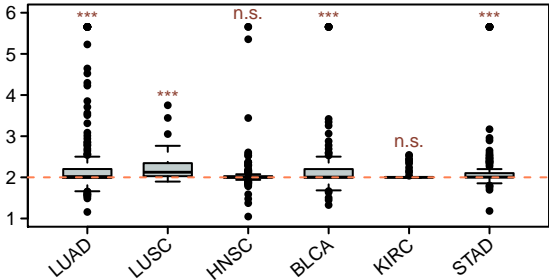

Supplement: S5 Fig — (PDF) [file pgen.1006994.s005.pdf]

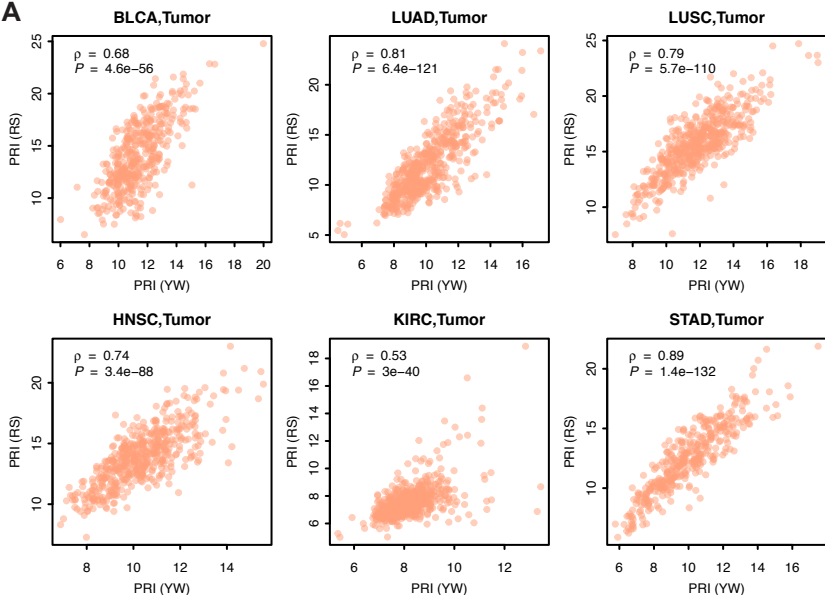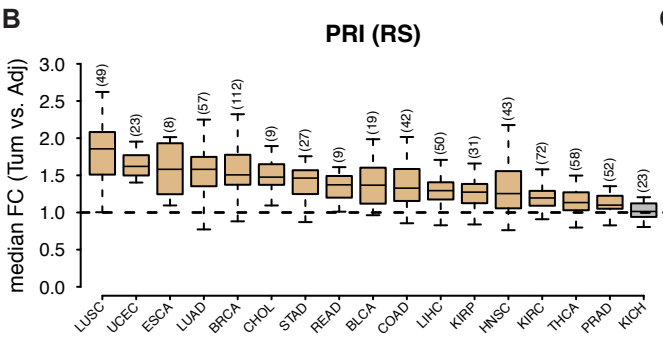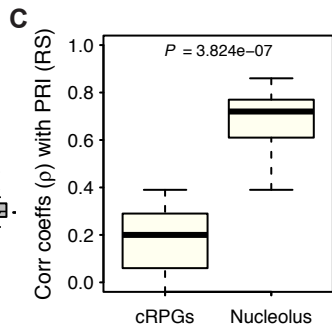

Supplement: S6 Fig — (A) PRIs calculated from the YW and RS gene sets are strongly correlated. (B) All cancer types have increased PRI except KICH. Seventeen cancer types with RNA-seq data in ≥ 5 tumor-adjacent pairs were shown, with sample sizes in brackets. Yellow and grey indicate significant up-regulation (Wilcoxon rank sum test P < 0.01) and not significant in tumors compared with paired adjacent controls, respectively. (C) The Spearman’s correlation coefficients of PRI with nucleolar genes are significantly higher than that with cRPGs for the 17 cancer types (P from paired Wilcoxon rank sum test). RS set was used in B and C. COAD, colon adenocarcinoma; KIRP, kidney renal papillary cell carcinoma; THCA, thyroid carcinoma; READ, rectum adenocarcinoma; KICH, kidney chromophobe; PRAD, prostate adenocarcinoma; CHOL, cholangiocarcinoma; UCEC, uterine corpus endometrial carcinoma; ESCA, esophageal carcinoma. Other abbreviations are as in Table 1. (PDF) [file pgen.1006994.s006.pdf]

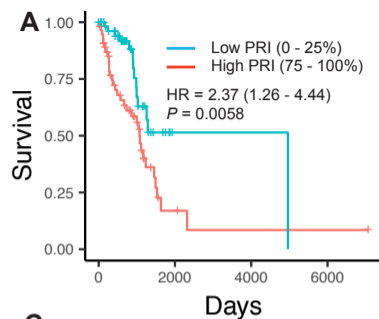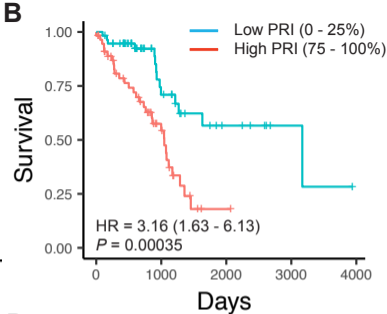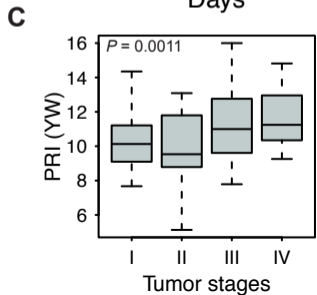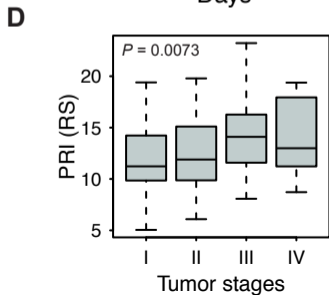

Supplement: S7 Fig — Example using LUAD data show that patients with higher PRI had (A, B) worse survival (comparing the last with the first 25% patients, logrank test, P < 0.006, Hazards ratio > 2.35), as well as (C, D) more severe tumor stage (ANOVA, P < 0.0075). The YW gene set is used for A and C; while the RS gene set is used for B and D. (PDF) [file pgen.1006994.s007.pdf]

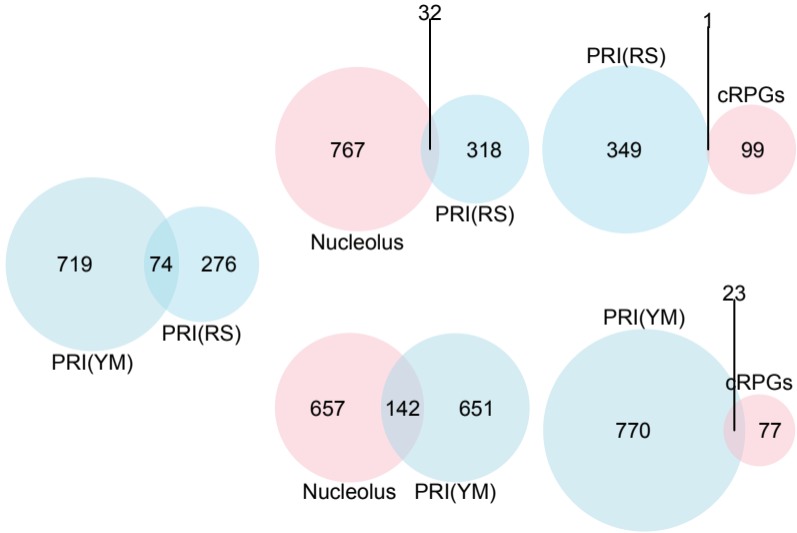

Supplement: S8 Fig — Note that the gene-set used to calculate PRI is mostly distinct from the gene-set used to calculate nucleolar activity. (PDF) [file pgen.1006994.s008.pdf]

**A**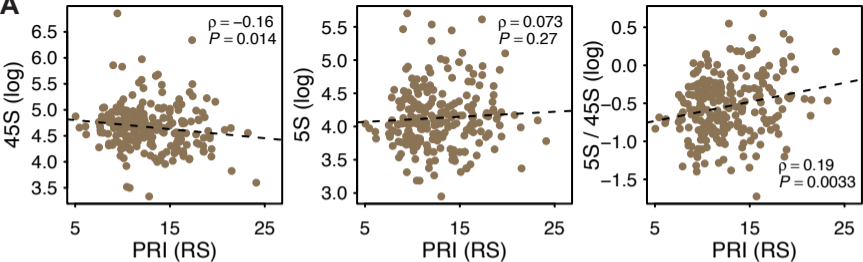**B**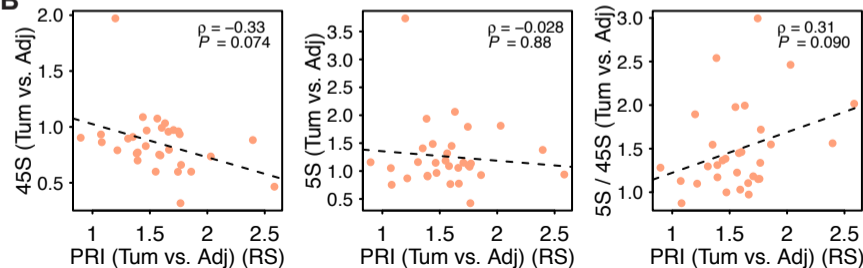

Supplement: S9 Fig — (A) PRI is significant negatively correlated with 45S and positively with the 5S / 45S ratio whereas it is not significant with 5S in tumors. (B) Consistent results were observed when associating tumor vs. adjacent normal fold change of PRI with that of 5S, 45S or their ratio for 31 patients. RS set genes and LUAD samples are used. (PDF) [file pgen.1006994.s009.pdf]

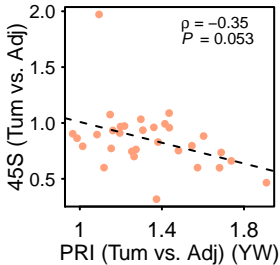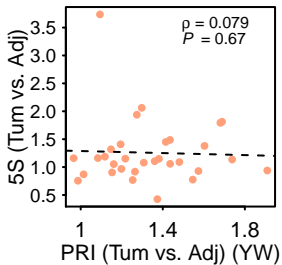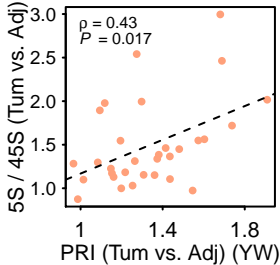

Supplement: S10 Fig — Spearman correlations between relative fold change of proliferation in tumor relative to its adjacent, and the fold change of 5S, 45S or their ratio for same patients. LUAD samples are used. (PDF) [file pgen.1006994.s010.pdf]
